# The neglected challenge: Vaccination against rickettsiae

**DOI:** 10.1371/journal.pntd.0008704

**Published:** 2020-10-22

**Authors:** Anke Osterloh

**Affiliations:** Research Center Borstel, Schleswig-Holstein, Germany; University of Oxford, UNITED KINGDOM

## Abstract

Over the last decades, rickettsioses are emerging worldwide. These diseases are caused by intracellular bacteria. Although rickettsioses can be treated with antibiotics, a vaccine against rickettsiae is highly desired for several reasons. Rickettsioses are highly prevalent, especially in poor countries, and there are indications of the development of antibiotic resistance. In addition, some rickettsiae can persist and cause recurrent disease. The development of a vaccine requires the understanding of the immune mechanisms that are involved in protection as well as in immunopathology. Knowledge about these immune responses is accumulating, and efforts have been undertaken to identify antigenic components of rickettsiae that may be useful as a vaccine. This review provides an overview on current knowledge of adaptive immunity against rickettsiae, which is essential for defense, rickettsial antigens that have been identified so far, and on vaccination strategies that have been used in animal models of rickettsial infections.

## Introduction

Rickettsioses are emerging febrile infectious diseases that are caused by small obligate intracellular bacteria of the family of *Rickettsiaceae* that consists of 2 genuses, *Rickettsia* and *Orientia*. The genus *Orientia* contains at least 2 closely related members, *Orientia tsutsugamushi* and *Orienta chuto* [1] and maybe further, so far unnamed, novel *Orientia* species [2]. The genus *Rickettsia* is further divided into 3 major groups of pathogenic bacteria: the spotted fever group (SFG), the typhus group (TG), and the transitional group. The majority of rickettsiae, approximately 20 species, belongs to the SFG that also causes the majority of infections worldwide; prominent members being *Rickettsia rickettsii*; the causative agent of Rocky Mountain Spotted Fever (RMSF); *Rickettsia conorii* that causes Meditarranean Spotted Fever; *Rickettsia africae* that is the etiologic agent of African Tick Bite Fever (ATBF); and *Rickettsia parkeri*. *Rickettsia prowazekii* and *Rickettsia typhi* are the 2 TG members and the causative agents of epidemic and endemic typhus, while *Rickettsia felis*, *Rickettsia akari*, and *Rickettsia australis* build the transitional group of pathogenic rickettsiae.

Rickettsiae are zoonotic pathogens that are transmitted to humans by the bite of ticks or via the feces of infected lice and fleas, while *Orientia* species are transmitted by chiggers. Except for *R*. *prowazekii*, which is transmitted from human to human via the body louse, rodents commonly serve as natural reservoirs for the bacteria [3]. However, transmission of *Orientia* from rodents has not been formally proven. Some SFG rickettsiae (e.g., *R*. *conorii*) and *O*. *tsutsugamushi* usually cause a characteristic eschar at the site of entry, while an eschar is not observed in the infection with *R*. *rickettsii* and TG rickettsiae. Further symptoms of disease, which are noticeable after approximately 7 to 14 days, are quite unspecific. With the onset of disease, patients commonly present with high fever, and a high percentage shows a characteristic spotted skin rash on days 3 to 5 of fever, which is caused by local bleedings and inflammatory reactions. In the initial phase immediately after entering the body, rickettsiae first infect phagocytic cells in the skin and then spread into endothelial cells (ECs) that build the inner wall of the blood vessels [4,5], where they replicate free in the cytosol. SFG rickettsiae can spread from cell to cell driven by actin-based motility without destroying the cell [6–8], while TG rickettsiae are considered to accumulate in the cell until lysis [6]. In contrast, *Orientia* has been shown to induce a kind of budding and leaves infected cells coated by cellular membrane [9]. After release, the bacteria spread into adjacent cells and distribute in the body via the blood stream. Rickettsiae can then enter nearly all tissues and organs and infect other cells, especially monocytes/macrophages (MΦ) [4,5,10] that are also proposed to serve as a replicative niche [11] and as a vehicle for the transport of the bacteria for dissemination through the blood [12,13]. In vitro, rickettsiae can infect nonimmune cells including hepatocytes [14,15], smooth muscle cells [16], neurons [17], and fibroblasts. The latter are also commonly used for the in vitro culture of the bacteria for research and diagnostic purposes [18–21]. Whether neurons or fibroblasts can get infected in vivo is not clear. Infection of these cells in patients has not been observed.

Rickettsiae systemically spread in the body and can cause multi-organ failure with fatal outcome. Complications that are often observed in severe cases of rickettsial infections are interstitial pneumonia, meningoencephalitis or meningitis, myocarditis, nephritis, and liver necrosis [22,23]. However, depending on the rickettsial species, the severity of disease and lethality strongly varies. The highest lethality is observed for the infection with *R*. *rickettsii*, nowadays 1%–7% [24] and 23% in the preantiobic era, and for the infection with *R*. *prowazekii* (15% up to 30% under circumstances of poverty, starvation, and lack of nursing care [23,25,26]). For that reason, these rickettsial species are classified as potential bioweapons.

With regard to the potential use of *R*. *prowazekii* and *R*. *rickettsii* as bioweapons, it is important to mention that genetic manipulation of these agents to acquire antibiotic resistance is possible. Meanwhile, genetic engineering has been shown for several rickettsial species [27–32]. In addition, targeted gene knockout by homologous recombination was successful for *R*. *prowazekii* [33] and for *R*. *rickettsii* employing a targetron plasmid vector [34]. Similar genetic techniques may be used to introduce factors that enhance the pathogenicity of these potential bioweapons.

Apart from the consideration of rickettsiae as potential bioweapons, rickettsioses are quite common but still neglected and underdiagonized diseases that predominantly affect people in poor countries where standards of hygiene are low. So far, the presence of *O*. *chuto* is considered to be restricted to the United Arab Emirates [1,3], while *O*. *tsutsugamushi* is common in the Asia-Pacific region reaching from southern parts of eastern Russia, over Japan to China, India, Pakistan, Indonesia, the north of Australia, and Afghanistan [35]. Scrub typhus is the most common rickettsiosis in India [35]. *R*. *typhi* generally occurs worldwide and is also endemic in Asia. Typhus, most likely the infection with *R*. *typhi* rather than *R*. *prowazekii*, is a serious threat to public health in China, mainly northern China [36], where farmers and the elderly are at enhanced risk [37]. Both *O*. *tsutsugamushi* and *R*. *typhi* have just recently been recognized to be major causative agents of severe meningitis and meningoencephalitis with high lethality rates [38]. Moreover, rickettsial infections generally occur worldwide with increasing incidence and geographic expansion. Scrub typhus is reemerging in southern China where an exponential increase in the incidence and geographic extension of the disease is observed since 2006 [39]. Similar is true for South Korea where 70,914 cases of Scrub typhus were reported by the Korea Center for Disease Control during 2001 to 2013, while no autochthonic cases were recorded from 1951 to 1985 [40,41]. In addition, *O*. *tsutsugamushi* was recently recognized in Chile [42], some countries of Africa, namely Kenia [43,44] and Senegal [45], as well as in France where rodents were found to be positive for *O*. *tsutsugamushi* [45]. Similar is true for spotted fever rickettsioses (SFRs). A total of 495 cases of SFR were recorded by the Centers for Disease Control and Prevention (CDC) in 2000, while around 5,500 cases were reported in 2018. It is unclear, however, how many of these cases are RMSF caused by *R*. *rickettsii* or other spotted fevers (https://www.cdc.gov/rmsf/stats/index.html). RMSF is also reemerging after decades of quiescence in Mexico [46], Panama [47–49], Colombia [50,51], and Brazil [52–54]. Also, steadily increasing case numbers and geographic expansion of *R*. *typhi* infections are recognized in the United States of America. While 27 cases were reported to the Texas State Health Department in 2003, 738 cases were confirmed in 2018 [55] (https://www.dshs.texas.gov/IDCU/disease/murine_typhus/Statistics.aspx). In addition, sporadic cases of epidemic typhus are reported from several states of eastern US in recent decades (https://www.cdc.gov/typhus/epidemic/), which has been associated with contact to flying squirrels that are considered a natural reservoir for *R*. *prowazekii* [56,57].

The spectrum of drugs for the therapy of rickettsial infections is currently still limited. Antibiotics that are active against rickettsiae are tetracyclines (e.g., doxycycline), macrolides (e.g., azithromycin) or chloramphenicol that inhibit the ribosomal biosynthesis of proteins, and rifamycins (e.g., rifampin) that inhibit the bacterial RNA polymerase, and thus, RNA transcription. Doxycycline is the antibiotic of choice for the treatment of all rickettsial infections.

The fact that rickettsiae respond to only few antibiotics bares 4 problems: (1) The unspecific symptoms of rickettsial infections often lead to misdiagnosis and in the following to the treatment with inappropriate antibiotics and to disease progression to a more severe outcome. (2) Some people are doxycycline intolerant. In this case, alternatives are rare. Rifampin has been successfully used for the treatment of ATBF in a patient with doxycycline intolerance [58]. This antibiotic, however, is not considered an acceptable and appropriate treatment for RMSF [59], and it is questionable whether it is effective against other severe rickettioses such as epidemic typhus. (3) Apart from that, the risk of the development of resistance against these antibiotics is high. It has not only been shown that TG rickettsiae acquire resistance against rifampin in laboratory experiments [60,61] but also resistance against this antibiotic occurs in natural strains of SFG rickettsiae [62]. Similarly, resistance against doxycycline may be acquired. In areas where *O*. *tsutsugamushi* is endemic resistance against doxycycline has been reported [63–65]. True resistance of *Orientia* strains against doxycycline, however, is questioned because of the different methodology that was used in the few studies mentioned above and because the supposedly resistant bacteria could also result from a lack of response to treatment of the patient [66,67]. Standardized methods for the detection of resistance are still missing. (4) Finally, it is known that rickettsiae can persist despite the treatment with antibiotics. Persistent infections after antibiotic treatment are observed in the infection with *O*. *tsutsugamushi* [68], *R*. *rickettsii* [69,70], and *R*. *prowazekii* [71], and similar is assumed for *R*. *typhi*, the second TG member [13]. *R*. *prowazekii* can reoccur years to decades after primary infection, causing the so-called Brill Zinsser disease [72–75]. In addition, relapse of patients that had been treated with antibiotics and recovered from *O*. *tsutsugamushi* infection is observed months to years after primary infection [68]. Recurrence of other persisting rickettsial species cannot be excluded, although it has not been described or recognized yet. In this context, it is possible that treatment with wrong antibiotics or irresponsiveness of a patient to certain antibiotics may facilitate persistence.

For these reasons, apart from the development of new drugs for therapeutic treatment of the infection, vaccines that can prevent rickettsial infections are urgently needed and would be beneficial in endemic areas as well as for travelers in these regions.

While innate immune responses are clearly important in early defense against rickettsial infections [4,76,77], adaptive immunity is essential for protection. Vaccine development requires the understanding of protective adaptive immune responses against rickettsiae, the identification of immunogenic rickettsial antigens, and strategies to target and direct protective immune responses. This review provides a short overview on adaptive immunity against rickettsial infections and mainly focuses on the efforts and progresses that have been made to identify immunogenic targets and vaccine candidates.

## Adaptive immunity against rickettsiae

**Fig 1:**
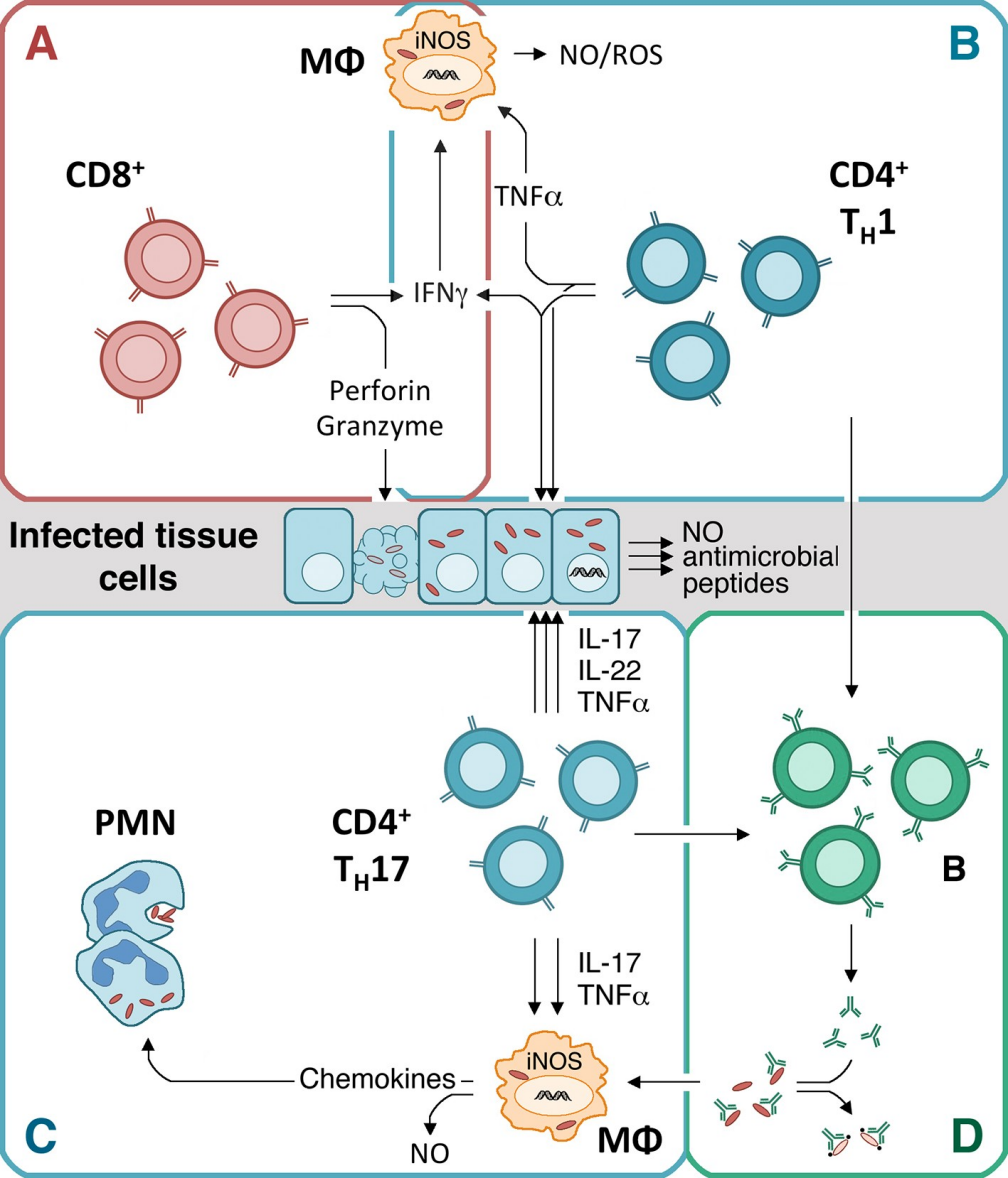
Immune response against rickettsiae. CD8^+^ cytotoxic T cells play the most important role against most rickettsiae and can directly kill infected cells. Apart from the cytotoxic activity, CD8^+^ T cells also release IFNγ. The cytotoxic activity of CD8^+^ T cells plays a dominant role in defense against SFG rickettsiae and *Orienta*, while the release of IFNγ seems to be more important in long-term control of TG rickettsiae (A). IFNγ, which is released at high amounts by CD4^+^ T_H_1 cells in addition to TNFα, acts against rickettsiae by activating antimicrobial mechanisms, e.g., NO production in MΦ and other infected cells (B). In the absence of IFNγ, CD4^+^ T cells develop into T_H_17 cells that produce IL-17, IL-22, and TNFα. These cells can also protect against rickettsial infections by acting on MΦ via IL-17 and TNFα that induce the production of NO and the release of chemokines that attract neutrophils (PMNs). IL-22, in addition to IL-17 and TNFα, also induces the production of NO, antimicrobial peptides, and other microbicidal mechanisms in infected tissue cells. In this way, T_H_17 cells are capable to eliminate the bacteria. The combined release of TNFα and IL-17, however, exerts pathological effects (C). The production of specific high-affinity antibodies by B cells depends on T cell help. Specific antibodies are produced late in the infection with rickettsiae and are considered to play a minor role in primary defense. Antibodies can contribute to defense most likely by opsonizing the bacteria for the uptake and destruction by MΦ or the activation of complement to mediate direct bacterial killing (D). The most promising way to achieve immunity against rickettsiae by a vaccine is the induction of specific cytotoxic CD8^+^ and/or IFNγ-producing CD4^+^ T_H_1 cells, in best case in addition to antibody-producing B cells. IFNγ, interferon gamma; IL-17, interleukin 17; IL-22, interleukin 22; iNOS, inducible nitric oxide synthase; MΦ, monocytes/macrophages; NO, nitric oxide; PMN, polymorphonuclear neutrophils; ROS, reactive oxygen species; SFG, spotted fever group; TG, typhus group; TNFα, tumor necrosis factor alpha.

Several studies in murine models of the infection with rickettsiae have clearly demonstrated that adaptive immunity is essential for protection. This is clearly reflected by the fact that T and B cell–deficient mice are highly susceptible to the infection with TG as well as SFG rickettsiae [13,78,79]. The current knowledge on the role of B and T cells in defense against rickettsiae is summarized in the following paragraphs and depicted in Fig 1.

### Antibody-mediated immunity and B cell antigens

Antibodies produced by B cells can generally contribute to protection against invading pathogens by different mechanisms that depend on the constant fragment crystallizable (Fc) part: (1) the opsonization of particles, which enhances the uptake and destruction by phagocytes; (2) the activation of complement after binding to the pathogens surface, which can directly kill the pathogen; and (3) in case of intracellular pathogens, the inhibition of cellular entry by binding to surface molecules that are essential for cell surface receptor binding and uptake.

In rickettsial infections, it is assumed that antibodies play a minor role in defense in primary infection because specific high-affinity antibodies that are produced with the help of CD4^+^ T cells are generated quite late in the infection. In humans, serum conversion in the infection with *R*. *typhi* takes place around day 15 after the onset of symptoms [80] and even later in the infection with *R*. *conorii* and *R*. *africae* (day 16 and day 25) [81]. In experimental animal models, antibodies to rickettsiae appear after recovery of the animals. Therefore, it is unlikely that they contribute to clearance in primary infection. Nonetheless, antibodies act protectively and may help to prevent or ameliorate secondary infections. Passive immunization of C3H SCID mice with polyclonal immune serum from *R*. *conorii*-infected mice protected the mice against a lethal challenge with *R*. *conorii* and even led to prolonged survival and reduced bacterial load in already infected C3H SCID mice [79].

So far, it seems that there are only few dominant antigens that are recognized by potentially protective antibodies in infected individuals. Members of the “surface cell antigen” (Sca) autotransporter family (namely Sca0 to 5) clearly represent immunodominant antigens that are recognized by antibodies in the sera from experimentally infected animals as well as human patients [82]. Among these, outer membrane protein A (OmpA/Sca0) that is expressed by SFG but not by TG rickettsiae and OmpB (Sca5) that is expressed by all rickettsiae are the most prominent antigens. Except for Sca4, which is found in the cytosol of the bacteria [83], these high molecular weight proteins are expressed on the cell surface of rickettsiae where they are easily accessible for antibodies. Especially, OmpA and OmpB are considered pathogenicity factors. Both proteins have been shown to be involved in the adherence of rickettsiae to host cells. *Escherichia coli* bacteria expressing surface-exposed recombinant OmpB from *Rickettsia japonica* or *R*. *conorii* acquired properties to adhere to and invade nonphagocytic Vero and HeLa cells [84,85], which was dependent on the extracellular outer membrane-associated passenger domain of the protein (OmpB_36_-_1334_) [85]. In addition, antibodies directed against OmpA inhibited adherence of *R*. *rickettsii* to L929 cells in vitro [86].

It is therefore likely that antibodies against OmpA and/or OmpB can contribute to protection. Indeed, monoclonal antibodies directed against *R*. *rickettsii* (most likely recognizing OmpA and OmpB) protected mice from a lethal short-term challenge with a large and toxic dose of homologous bacteria [87–89] and prevented fever and rickettsemia in guinea pigs [89]. Furthermore, monoclonal antibodies that are directed against the extracellular passenger domain of *R*. *conorii* OmpB are sufficient for protection of C3H/HeN mice against a lethal challenge with *R*. *conorii* [90]. Finally, the application of polyclonal anti-*R*. *conorii* immune serum as well as monoclonal anti-*R*. *conorii* OmpA or anti-OmpB antibodies protected even immunodeficient C3H/HeN SCID mice against challenge with *R*. *conorii* [79].

The mechanism of protection by these antibodies is not clear, but there are hints that opsonization of the bacteria for the uptake by phagocytic cells rather than the inhibition of the binding of the bacteria to nonphagocytic host cells may play a role in bacterial defense. It was observed that the opsonization of *R*. *conorii* with either polyclonal or monoclonal antibodies against OmpA and OmpB leads to enhanced engulfment of the bacteria by ECs (SVEC4–10) and MΦ-like cells (J774A.1) in vitro [91]. In addition, bacterial growth in these cells was reduced [91]. Also, the treatment of C3H/HeN SCID mice with polyclonal antiserum resulted in enhanced killing of *R*. *conorii* by MΦ and the accumulation of rickettisal antigens in MΦ in the spleen [79]. More recent in vitro investigations indicate that monoclonal antibodies recognizing the extracellular passenger domain of OmpB also can induce complement-mediated killing of the bacteria [90], which has not been described before.

For *R*. *prowazekii*, 4 B cell epitopes of OmpB (OmpB_45-58_, OmpB_1239-1252_, OmpB_1259-1268_, and OmpB_1287-1296_) have been identified that are recognized by polyclonal antibodies from rabbits immunized with purified OmpB [92]. Three of these are also recognized by antisera from human patients (OmpB_45-58_, OmpB_1239-1252_, and OmpB_1287-1296_) [92]. In addition, different Sca-derived peptides (Sca1_753-665_, Sca2_496-509_, Sca3_314-327_, Sca4_263-276_, and OmpB (Sca5_651-665_)) from *R*. *typhi* that were fused to form multivalent antigens were found to induce antibody response upon immunization of rabbits [93]. Whether these antibodies have protective properties, however, is unknown.

Apart from the high molecular weight OmpA and OmpB proteins, additional immunodominant proteins are recognized by antibodies from infected individuals. One of these is the 60 heat shock protein GroEL. GroEL was found to be the most prominent antigen from *R*. *conorii* that is recognized by antibodies in sera from immunized rabbits as well as from infected patients [94]. Similar is true for GroEL from *Rickettsia heilongjiangensis*, *Rickettsia helvetica*, *and R*. *parkeri* that is recognized by antibodies in the sera from patients as well as mice infected with these pathogens [94–97]. GroEL acts as a chaperone that assists in the folding of proteins in the cytosol of prokaryotice cells and is up-regulated under circumstances of stress. For *R*. *prowazekii*, it was shown that GroEL is up-regulated in the early phase of infection [98] where enhanced chaperone activity may be necessary. Despite its cytosolic chaperone function, however, GroEL appears in multiple isoforms in rickettsiae and has been shown to be surface exposed in *R*. *conorii* [94] and *R*. *heilongjiangensis* [95]. A potentially protective function of antibodies against GroEL, however, has not been investigated yet, but it is interesting that GroEL is considered a promising vaccine candidate for other bacterial infections such as *Mycobacterium tuberculosis* [99], *Bacillus anthracis* [100], and *Helicobacter pylori* [101,102].

Other immunodominant proteins that are recognized by antibodies in the infection with *R*. *heilongjiangensis* are PrsA, RplY, RpsB, SurA, and YbgF [95] and Sta22, Sta47, Sta56, ScaA, and ScaC in the infection with *O*. *tsutsugamushi* [103–107]. PrsA, a Parvulin-like peptidylprolyl isomerase, presumably assists in the folding of periplasmatic and membrane proteins and is likely expressed in the outer membrane of the bacteria. RplY and RpsB are ribosomal proteins that are most likely expressed in the cytosol of the bacteria. The same is true for SurA, another peptidylproly isomerase that acts as a chaperone. YbgF belongs to the Tol/Pal-system of bacteria and is involved in the maintenance of the membrane integrity of the outer bacterial membrane and can be surface exposed. ScaA, Sta56, and ScaC from *O*. *tsutsugamushi* are also proteins of the outer membrane, while Sta22 and St47 are considered to locate in the cytosol, cytosolic membrane, or periplasma. ScaA has been shown to be involved in adhesion of the bacteria to nonphagocytic HeLa cells, which is blocked by anti-ScA antibodies but not by antibodies against ScaB, ScaC, or ScaE [108]. Similarly, antibodies against recombinant Sta56 from the *O*. *tsutsugamushi* Boryong strain that were produced in mice and rabbits inhibit adhesion and infection of L929 cells by *Orientia* in vitro [109], and certain monoclonal Sta56-specific antibodies were protective against challenge of mice with the homologous *O*. *tsutsugamushi* strain in vivo [110]. The role and mode of action of antibodies against these antigens in defense against rickettsiae in vivo, however, is still not clear and remains to be investigated.

### T cell–mediated protection against rickettsiae

It is clearly undisputed that T cells rather than B cells play a critical role in protection against rickettsial infections. The most important role in protection is ascribed to cytotoxic CD8^+^ T cells that are capable of direct killing of infected cells. CD4^+^ T cells on the other hand can contribute to protection by the release of effector molecules that can activate phagocytes and by driving the activation of B cells to produce specific antibodies. CD8^+^ as well as CD4^+^ T cells have been shown to be involved in protection against rickettsial infections in animal model systems. In recent years, however, it is emerging that the importance of CD8^+^ and CD4^+^ T cell populations in defense against SFG and TG rickettsiae may differ. The following paragraph summarizes current knowledge on the role of CD8^+^ and CD4^+^ T cells in protection against SFG, TG, and transitional rickettsiae and *Orientia*.

### Role of CD4^+^ T cells and CD8^+^ T cells in defense against SFG and transitional rickettsiae

In the experimental infection of C3H/HeN mice with *R*. *conorii* (SFG) and *R*. *australis* (transitional group), a peak response of activated CD8^+^ T cells that release interferon gamma (IFNγ) and exert enhanced cytotoxic function is observed at day 10 postinfection [111]. Experimental animal models of the infection with these bacteria further indicate that CD8^+^ T cells are essential for defense against these pathogens. C3H/HeN mice that were depleted of CD8^+^ T cells showed reduced survival, increased bacterial burden, and enhanced pathology in the infection with a sublethal dose of *R*. *conorii* [111,112]. Furthermore, immune CD8^+^ T cells adoptively transferred into C3H/HeN mice protected the animals against infection with a normally lethal dose of *R*. *conorii* [112]. The importance of CD8^+^ T cells in defense against SFG rickettsiae is further evidenced by the enhanced susceptibility of C57BL/6 MHCI^-/-^ mice that lack CD8^+^ T cells to a lethal outcome upon infection with *R*. *australis* compared to wild-type mice [111]. The cytotoxic activity of CD8^+^ T cells rather than the release of IFNγ seems to be the main effector mechanism that acts against these bacteria. Firstly, the adoptive transfer of immune CD8^+^ T cells from C57BL/6 IFNγ^-/-^ mice into *R*. *australis*-infected C57BL/6 IFNγ^-/-^ mice led to reduced bacterial load and protection [111]. Secondly, C57BL/6 Perforin^-/-^ mice where CD8^+^ T cells lack the cytotoxic potential showed a higher susceptibility and lethality upon infection with *R*. *australis* compared to wild-type as well as to C57BL/6 IFNγ^-/-^ mice [111]. C57BL/6 Perforin^-/-^ mice, however, were less susceptible to *R*. *australis* than C57BL/6 MHCI^-/-^ that do not possess CD8^+^ T cells at all [111]. Together, these findings indicate that the cytotoxic function of CD8^+^ T cells plays a dominant role in defense against these bacteria compared to the release of IFNγ.

In contrast to the depletion of CD8^+^ T cells, the depletion of CD4^+^ T cells in C3H/HeN mice by administration of neutralizing antibodies altered neither the course of disease in the infection with a sublethal dose of *R*. *conorii* nor the bacterial load in different organs compared to control mice that received control antibodies. Both groups cleared the infection with similar kinetics [112]. Nonetheless, similar to the adoptive transfer of immune CD8^+^ T cells, the transfer of immune but not naive CD4^+^ T cells into C3H/HeN mice was protective against a normally lethal infection with *R*. *conorii* [112]. The data demonstrate that CD4^+^ T cells can contribute to defense against SFG rickettsiae, although CD8^+^ T cells obviously play a dominant role.

The main effector molecules that are considered to be involved in CD4^+^ T cell–mediated defense against intracellular pathogens are IFNγ and tumor necrosis factor alpha (TNFα). Both cytokines can contribute to the killing and elimination of intracellular agents by activating the bactericidal function of phagocytic and responsive nonphagocytic cells, namely the induction of the expression of inducible nitric oxide (NO) synthase (iNOS) and the production of NO [113–116].

IFNγ and TNFα have also been involved in defense against *R*. *conorii* and *R*. *australis*. Both cytokines induced bacterial killing in *R*. *conorii*-infected human cell lines in vitro, which was dependent on the production of NO [117]. The neutralization of either IFNγ or TNFα leads to reduced survival, overwhelming bacterial burden, and enhanced pathology in *R*. *conorii*-infected C3H/HeN mice, which was associated with reduced NO production [118]. Furthermore, IFNγ-deficient C57BL/6 mice succumb to the infection with a normally sublethal dose of *R*. *conorii* [118]. Finally, also IFNγ^-/-^ C57BL/6 mice showed reduced survival in the infection with *R*. *australis* [111].

Generally, C3H/HeN mice and C57BL/6 differ in their susceptibility to rickettsial infections [76]. One reason for that may be the different ability of dendritic cells (DCs) to induce protective immune responses. *R*. *conorii*-infected DCs from C3H/HeN mice are less effective in the in vitro induction of IFNγ production in CD4^+^ T cells than DCs from C57BL/6 mice, which can be ascribed to lower major histocompatibility complex II (MHCII) expression and reduced release of interleukin 12 (IL-12) [119]. In addition, higher frequencies of regulatory CD4^+^FoxP3^+^ T cells are observed in C3H/HeN compared to C57BL/6 mice in the infection with *R*. *conorii* [119]. In another study, it was shown that CD4^+^ T cells with an inducible regulatory phenotype (CD4^+^CD25^+^FoxP3^-^T-bet^+^CTLA4^high^) produced IFNγ and IL-10 in the infection of C3H/HeN mice with a lethal dose of *R*. *conorii* and that these cells suppressed proliferation and IL-2 release by CD4^+^ T cells in vitro [120]. These data suggest that immune suppression by regulatory T cells may contribute to enhanced susceptibility.

### Role of CD4^+^ T cells and CD8^+^ T cells in defense against TG rickettsiae

Corresponding to CD8^+^ T cells that develop in the infection with SFG rickettsiae, CD8^+^ T cells from *R*. *typhi*-infected BALB/c and C57BL/6 mice produce increased levels of IFNγ and Granzyme B upon in vitro restimulation with phorbol myristate acetate (PMA)/Ionomycin [121,122], indicating enhanced cytotoxic activity. A peak response of activated CD8^+^ T cells in the infection with *R*. *typhi* is observed at day 7 postinfection [121,122] and thus a little earlier than in the infection with SFG rickettsiae. It has been previously shown that *R*. *typhi* persists in these mouse strains [13] that are considered resistant to the infection as they do not develop symptoms of disease. In concordance with the presence of persisting bacteria, levels of activated CD8^+^ T cells do not decline to basal levels for a long period of time in BALB/c as well as in C57BL/6 mice in the infection with *R*. *typhi* [121,122]. Moreover, enhanced amounts of activated CD8^+^ T cells are detectable in *R*. *typhi*-infected BALB/c mice in periodic intervals [122]. These findings indicate that activated CD8^+^ T cells are of importance for the control of the persisting bacteria. In line with that, it was found that the depletion of CD8^+^ T cells in the infection of C3H/HeN mice with *R*. *typhi* leads to enhanced bacterial burden and pathology [123].

For a more detailed study of the role and function of CD8^+^ (and CD4^+^ T cells) in protection against *R*. *typhi*, 2 animal models have been developed in recent years: immunodeficient C57BL/6 RAG1^-/-^ and BALB/c CB17 SCID mice, both of which lack adaptive immunity but behave differently in the infection. C57BL/6 RAG1^-/-^ mice can control the bacteria for approximately 3 months. Then, the bacteria suddenly start to grow more or less exclusively in the brain. As a consequence of massive inflammation of the central nervous system, the animals become ataxic and paralyzed and finally die [13]. The course of disease in BALB/c CB17 SCID completely differs from that. These animals show high bacterial burden in all organs, develop liver necrosis and splenomegaly, and die from high systemic inflammation within 3 weeks [78].

In the C57BL/6 RAG1^-/-^ model, the adoptive transfer of immune CD8^+^ T cells was 100% protective against *R*. *typhi* even when transferred late in the infection shortly before the onset of disease [121]. Similarly, the adoptive transfer of naive CD8^+^ into BALB/c CB17 SCID mice prior to the infection with *R*. *typhi* was protective and none of the animals developed disease or died [122]. CD8^+^ T cells, however, do not require cytotoxic activity to act against *R*. *typhi*. BALB/c Perforin^-/-^ mice where CD8^+^ T cells lack the cytotoxic function are not susceptible to *R*. *typhi* [122]. Again, this is in contrast to the enhanced susceptibility of C57BL/6 Perforin^-/-^ mice to the infection with *R*. *australis* [111]. Moreover, the adoptive transfer of CD8^+^ Perforin^-/-^ T cells still protected BALB/c CB17 SCID from *R*. *typhi* infection. As in the transfer of immunocompetent CD8^+^ T cells, the mice did not even show symptoms of disease at any point in time [122]. Thus, the cytotoxic activity of CD8^+^ T cells is not essential for protection in the infection with *R*. *typhi*.

Further data show that the lack of the cytotoxic activity of CD8^+^ T cells can be compensated by the release of IFNγ and vice versa. First of all, BALB/c IFNγ^-/-^ mice are as resistant to *R*. *typhi* as BALB/c Perforin^-/-^ mice. Furthermore, the adoptive transfer of CD8^+^ IFNγ^-/-^ T cells into *R*. *typhi*-infected BALB/c CB17 SCID mice was as protective as the transfer of CD8^+^ Perforin^-/-^ T cells [122]. In contrast to the infection with *R*. *australis* where the release of IFNγ by CD8^+^ T cells was found to be not essential for protection [111], IFNγ was even more important than the cytotoxic activity of CD8^+^ T cells in long-term control of *R*. *typhi*. Persisting bacteria were not found in BALB/c CB17 SCID mice that received CD8^+^ Perforin^-/-^ T cells but were detectable by quantitative polymerase chain reaction (qPCR) in CD8^+^ IFNγ^-/-^ T cell recipients, predominantly in the brain [122]. In contrast to the infection with *R*. *australis*, these data suggest that CD8^+^ T cells can protect against *R*. *typhi* either via cytotoxic activity or the release of IFNγ.

Overall, CD8^+^ T cells clearly confer protection against the infection with *R*. *typhi*. This is also demonstrated by the fact that C57BL/6 MHCII^-/-^ mice that lack CD4^+^ T cells are resistant against *R*. *typhi* and do not develop disease [121]. However, resistance against *R*. *typhi* was also demonstrated for C57BL/6 MHCI^-/-^ mice that lack CD8^+^ T cells [121]. This is in contrast to the infection of the same mice with *R*. *australis* where the lack of CD8^+^ T cells results in reduced survival [111]. These findings indicate that CD8^+^ T cells clearly play a dominant role in protection against *R*. *australis*, while either CD8^+^ or CD4^+^ T cells are sufficient for defense against *R*. *typhi*.

That CD4^+^ T cells alone are sufficient to mediate protection against the infection with *R*. *typhi* is further demonstrated by adoptive transfer of immune CD4^+^ T cells into susceptible T and B cell–deficient C57BL6 RAG1^-/-^ mice. Here, adoptively transferred immune CD4^+^ T cells still protected the mice even when transferred late in the infection when the bacteria already start to grow [121]. Furthermore, the adoptive transfer of naive CD4^+^ T cells, even at low amounts (1 × 10^6^), protected BALB/c CB17 SCID mice against challenge with *R*. *typhi* [121,122]. In both systems, CD4^+^ T cells were capable to eliminate the bacteria below qPCR detection limit, although CD8^+^ T cells were obviously more efficient and quicker in bacterial clearance. CD4^+^ T cells from C57BL/6 as well as from BALB/c mice express huge amounts of IFNγ and lower amounts of TNFα in the infection with *R*. *typhi* with a peak response around day 7 postinfection, which is similar to the CD8^+^ T cell response [121,122]. Also, the CD4^+^ T cell response does not return to basal levels [121,122], and CD4^+^ T cells are sporadically reactivated in *R*. *typhi*-infected BALB/c mice as observed for CD8^+^ T cells [121,122]. IFNγ as well as TNFα are activators of phagocytes such as MΦ and other cells and play an important role in defense against *R*. *conorii* [118]. In line with that, immune CD4^+^ T cells act on MΦ in the infection with *R*. *typhi* and activate the bactericidal activity of these cells via the release of IFNγ and TNFα [121,122]. Similar to the killing of *R*. *conorii*, IFNγ and TNFα induce the production of NO and bacterial killing of *R*. *typhi* in murine MΦ [122]. IFNγ was also shown to inhibit the growth of *R*. *prowazekii* in murine and human fibroblasts [124]. Nonetheless, the adoptive transfer of CD4^+^ T cells from BALB/c IFNγ^-/-^ mice into *R*. *typhi*-infected BALB/c CB17 SCID mice still protected a high percentage of the mice from a lethal outcome [122]. In this setup, CD4^+^ T cells developed into T_H_17 cells that produced high amounts of IL-22 and IL-17 in addition to lower amounts of TNFα [122]. Thus, T_H_1 as well as T_H_17 cells can be protective. For the latter, it has also been shown by neutralization experiments in vivo that the combined release of IL-17 and TNFα has immunopathological effects, while the presence of one or the other cytokine together with IL-22 is beneficial [122]. These data suggest that the induction of specific CD4^+^ T cells, especially IFNγ-producing T_H_1 cells, could be sufficient for protection against *R*. *typhi*.

### Role of CD4^+^ T cells and CD8^+^ T cells in defense against *Orientia*

T cells also play a dominant role against Orientia that resembles *Rickettsia* species with regard to lifestyle and targeting of ECs. It was first observed that BALB/c mice that recovered from an infection with the Gilliam strain of *O*. *tsutsugamushi* resist a normally lethal infection with the more virulent Karp strain of *O*. *tsutsugamushi* [125]. It was then further demonstrated that the adoptive transfer of nonadherent spleen from mice that recovered from the *O*. *tsutsugamushi* Gilliam infection conferred protection against lethal challenge with *O*. *tsutsugamushi* Karp [126]. The protective effect was clearly dependent on T cells, because mice that received immune spleen cells depleted of T cells were not protected anymore, while the depletion of B cells had no effect [126].

In more recent studies, it becomes clear that CD8^+^ T cells play a more critical role than CD4^+^ T cells in defense against Orientia. The infection of humans with *Orientia* leads to a loss rather than an increase in peripheral CD4^+^ T cells including regulatory T cells (Tregs) in the acute phase of infection, while activated CD8^+^ T cells increase [127]. Similarly, intravenous or subcutaneous infection of C57BL/6 mice as well as subcutaneous infection of BALB/c mice with *O*. *tsutsugamushi* Karp leads to a much stronger increase of CD8^+^ T cells than CD4^+^ T cells within the first 14 days after infection [128,129]. Furthermore, the CD8^+^ T cell response lasts for a long period of time (at least 135 days) in C57BL/6 mice [128]. In the subcutaneous infection model as well as in the intravenous infection, the bacteria spread to nearly all organs with highest bacterial loads in the lung [129,130], which is accompanied by an increase of CD8^+^ T cell infiltrates within the third weak after infection [128]. The prominent role of CD8^+^ T cells in defense was further demonstrated by the study of mice that were either depleted of CD8^+^ T cells or CD8^+^ T cell deficient as well as by the adoptive transfer of CD8^+^ versus CD4^+^ T cells. Depletion of CD8^+^ T cells in *O*. *tsutsugamushi*-infected BALB/c mice results in uncontrolled bacterial growth and death of the animals [128]. The same is true for the infection of CD8^+^ T cell–deficient C57BL/6 mice, either infected intravenously or via the skin. These mice show increasing bacterial loads in lung, kidney, liver, and spleen; more severe lesions in the organs; and die through a normally sublethal infection with *O*. *tsutsugamushi* [128,129]. The adoptive transfer of CD8^+^ T cells from immune BALB/c mice that recovered from the sublethal skin infection protected animals that were challenged with the homologous strain via the normally lethal intraperitoneal route [128]. The same observations were made when immune CD8^+^ T cells were transferred into C57BL/6 mice that were intravenously infected with a lethal dose of *Orientia* [129]. The long-lasting CD8^+^ T cell response seems to be associated with the persistence of the bacteria that has been described for humans [68,131] as well as for mice [132,133], because the depletion of CD8^+^ T cells in *O*. *tsutsugamushi*-infected C57BL/6 at day 84 postinfection leads to reactivation of the bacteria [128]. The protective effect of CD8^+^ T cells seems to rely on the cytoxic activity of these cells rather than cytokine production. This is evidenced by the observation that Perforin^-/-^ C57BL/6 mice die through the infection during the first 14 days, similar to CD8^+^ T cell–deficient mice, and show enhanced bacterial burden in several organs [128]. These studies demonstrate that CD8^+^ T cells seem to be indispensable for protection against *Orientia*.

Nonetheless, CD4^+^ T cells also contribute to protection. While the transfer of immune CD8^+^ T cells is 100% protective in the intravenous C57BL/6 infection model, the transfer of CD8^+^ T cell–depleted immune spleen cells still protects approximately 50% of the animals. In addition, the onset of disease in these mice is delayed [129]. Although B cells were present in the cell preparation used for transfer, it is likely that this protective effect can be largely ascribed to CD4^+^ T cells, most probable T_H_1 cells that produce IFNγ and TNFα. These cytokines are induced in CD4^+^ T cells by *Orientia*-infected DCs in vitro [134] and are considered to contribute to protection against *Orientia* by the mechanisms mentioned earlier. In line with that, the adoptive transfer of an IFNγ-producing T cell line generated from immune BALB/c mice after sublethal infection with *O*. *tsutsugamushi* Gilliam conferred protection against lethal intraperitoneal challenge with the homologous strain [135]. Furthermore, in *O*. *tsutsugamushi*-infected BALB/c and C57BL/6 mice, the bacteria were predominantly found in MΦ, and inflammatory iNOS-expressing MΦ infiltrates were detectable in the organs [128,130]. The mice produced enhanced levels of IFNγ that clearly contributed to the inhibition of bacterial growth in an iNOS-dependent fashion [128].

However, CD4^+^ as well as CD8^+^ T cells may also contribute to pathology. Xu and colleagues observed that the expression of IFNγ and Granzyme B as well as of TNFα and monocyte chemoattractant protein-1 (MCP-1) was enhanced in CD8^+^ T cell- and MHCI-deficient *Orientia*-infected C57BL/6 mice [129]. In addition, these mice showed more severe liver and kidney damage. Similarly, *O*. *tsutsugamushi*-infected BALB/c mice depleted of CD8^+^ T cells showed enhanced serum levels of IFNγ and stronger MΦ responses in liver and lung with an increase of these cells as well as an increase in iNOS expression [128]. These findings indicate that the absence of CD8^+^ T cells probably leads to enhanced activation of CD4^+^ T cells and cytotoxic natural killer (NK) cells with enhanced IFNγ production as a compensatory mechanism. Such enhanced inflammatory response can result in more severe pathology as observed in the infection with *R*. *typhi* upon the adoptive transfer of immune CD4^+^ T cells into C57BL/6 RAG1^-/-^ mice where CD4^+^ T cells, when transferred late in the infection, promote MΦ-mediated inflammation in the brain [121]. In this context, it is also interesting that elevated levels of IFNγ and TNFα are found in the peritoneal lavage of experimentally *O*. *tsutsugamushi*-infected C3H/HeN mice and BALB/c mice with higher levels in susceptible C3H/HeN mice compared to resistant BALB/c mice [136,137]. The infection of humans with *O*. *tsutsugamushi* is also associated with elevated serum levels of these cytokines in addition to other inflammatory cytokines, several chemokines, as well as of Granzymes A and B as indicators of the activation of cytotoxic CD8^+^ T and NK cells [138–142]. Although important for protection, a contribution of these mediators to pathology cannot be excluded.

Last but not least, human and mouse may differ in their immune response and susceptibility to the infection with *Orientia* and other pathogens. In contrast to mice, the longevity of immunity against *Orientia* in humans seems to be limited. CD4^+^ and CD8^+^ T cells that specifically react to membrane proteins of the bacteria decline in infected humans from 1 year after infection [143], which is different from the long-lasting T cell response in C57BL/6 mice [128], and it was suggested that this may be due to a lack of memory response. To achieve a better understanding of human immune response, Jiang and colleagues just recently tested a humanized mouse, the DRAGA mouse which is based on an immunodeficient mouse that was reconstituted with human hemotopoietic stem cells in the infection with *O*. *tsutsugamushi*. Footpad inoculation of *O*. *tsutsugamushi* Karp into these mice leads to the dissemination of the bacteria into various organs with highest bacterial loads in the lung as observed in infected BALB/c and C57BL/6 mice [128–130]. The humanized DRAGA mice develop splenomegaly and liver necrosis, and the infection is lethal in a dose-dependent manner, whereas C3H/HeJ or BALB/c mice that are infected via the same route survive the infection with the same dose [130,144]. A strong T_H_1 response with the production of high amounts of IFNγ, TNFα, IL-12, and IL-2 as well as an increase of activated human CD4^+^ and CD8^+^ T cells was observed in DRAGA mice. In addition, regulatory T cells and the production of IL-10 were significantly enhanced [144], which is also observed in the initial phase of the infection in C57BL/6 mice [129]. Overall, the infection and disease of these humanized mice largely resembles the infection of normal mice and humans. The expansion of CD8^+^ T cells, however, seems to be much more pronounced in normal mice as well as in humans compared to the DRAGA mouse.

### T cell–mediated cross-protection and T cell antigens

Animals as well as humans show cross-protective immune response against different rickettsial species. The experimental infection of guinea pigs with *R*. *typhi* renders the animals resistant to *R*. *prowazekii* and vice versa, and similar is true for humans [145]. Antigen preparations from *R*. *rickettsii*, *R*. *sibirica*, and *R*. *australis* but not *R*. *akari* produce reactivation of immune spleen cells from *R*. *conorii*-infected C3H/HeJ mice, while spleen cells from *R*. *akari*-immunized mice react to *R*. *conorii* and other SFG rickettsiae [146]. Furthermore, immunization of C3H/HeN mice with a sublethal dose of *R*. *conorii* and *R*. *australis* protects the animals against a lethal challenge with the heterologous pathogen [147]. These data demonstrate cross-immunity between SFG and transitional rickettsiae. Even cross-protection between SFG and TG rickettsiae has been described. C3H/HeN mice immunized with sublethal doses of *R*. *conorii* or *R*. *typhi* were protected against lethal challenge with one or the other rickettsial species [148]. Therefore, the identification of immunodominant T cell antigens that are present in a broader range of rickettsiae may lead to the identification of vaccine candidates that can confer protection against a broader range of rickettsial species.

The following paragraph summarizes the current knowledge on the efficiency of different immunization strategies and rickettsial T cell antigens identified so far.

## Immunization and rickettsial T cell antigens

### Vaccination with inactivated rickettsiae as whole cell antigen (WCA)

One of the first vaccines against rickettsial infection was developed by R. Spencer and R. Parker in 1924. The vaccine was produced by triturating *R*. *rickettsii*-infected ticks, which were produced in the laboratory by feeding them on infected guinea pigs [149]. The bacteria were then inactivated in phenol and formalin. Another early vaccine was produced in embryonated chicken eggs (*Cox* vaccine). Application of the inactivated *R*. *rickettsii Cox*-type vaccine prior to the onset of disease after naturally acquired infection reduces the severity of illness in man [150]. Both vaccines induce the production of antibodies [151]. When given 3 to 6 months before exposure to live bacteria, both vaccines, however, only lead to milder illness but do not prevent the disease [151]. Another vaccine against *R*. *rickettsii* was produced in embryonal chicken fibroblasts followed by formalin inactivation by the US military in the 1970s [152,153]. This formalin-killed vaccine applied 2 times completely protected cynomolgus monkeys (*Macaca fasciularis*) that were infected subcutaneaously 2 months after the last immunization with virulent *R*. *rickettsii* [154]. It also protected rhesus monkeys [155] and led to milder disease in human volunteers [156].

In the 1920s, R. L. Weigl produced a vaccine against epidemic typhus by intrarectal injection of *R*. *prowazekii* into lice that were fed on humans. The bacteria were then isolated by trituration of the intestinal canals of infected lice and inactivated in phenol. Two or 3 injections of this material protected guinea pigs [157]. This vaccine was also used during World War II to protect German soldiers from the disease [158]. An interesting note here is that the Weigl lab produced vaccine lots with different potency for protection of which the stronger ones were given to resistance fighters and the weaker ones to the German Army. In addition, Weigl smuggled the vaccine into ghettos, which was done under huge risk as the German forces monitored his work. At the same time, the US military also produced a vaccine against *R*. *prowazekii* and grew the bacteria in chicken egg yolk sacs. The formalin-inactivated bacteria were then used for the vaccination of US soldiers during World War II and led to a milder form of disease and protection against severe disease [159]. Further vaccines against epidemic typhus were developed by R. Castaneda and H. Zinsser who isolated *R*. *prowazekii* either from the lungs of intranasally infected rabbits (*Castaneda* vaccine) [159] or the tunica vaginalis and peritoneum of infected rats (*Zinsser-Castaneda* vaccine) followed by inactivation of the bacteria in formalin [160]. This vaccine protected guinea pigs when applied subcutaneously or intraperitoneally [161]. F. Veintemillas found that at least 3 doses of the Zinsser-Castaneda vaccine are needed to protect guinea pigs [162].

Similar attempts were made for the vaccination against *O*. *tsutsugamushi* with either formalin-fixed homogenized lungs from infected cotton rats [163,164] or formalin-killed purified *O*. *tsutsugamushi* [165]. Both vaccination strategies, however, were not effective in humans [165] and led to only limited protection against the homologous strain in mice [166,167]. In contrast to that, it was shown in a more recent study that immunization of C3H/HeN mice with formalin-killed *O*. *tsutsugamushi* protected the animals against challenge with the homologous strain and induced immunity that lasted longer than 8 months [168].

Overall, complete protection against *R*. *prowazekii*, *R*. *rickettsii*, or *O*. *tsutsugamushi* employing formalin-inactivated bacteria as a vaccine was generally not achieved in humans so far, which maybe ascribed to alterations in the antigenic determinants due to the fixation method.

Alternative inactivation methods to avoid possible losses in antigenicity are killing of the bacteria by heat (56°C) or irradiation. Vaccination with irradiated *O*. *tsutsugamushi* completely protected mice against challenge with the homologous strain [169–171], and in a very recent study, the vaccinating potential of heat-killed bacteria was analyzed in a canine model of RMSF. Dogs were immunized twice with heat-inactived *R*. *rickettsii* grown either in embryonated eggs or in Vero cells and then challenged with live *R*. *rickettsii* intravenously. This vaccine protected the dogs from severe RMSF and reduced tissue lesions [172].

Together, these findings indicate that the method of inactivation for vaccine preparation plays a critical role for the protective potential.

#### Vaccination with live avirulent or attenuated rickettsiae

Apart from the immunization with intact but inactivated rickettsiae, other approaches employed live bacteria for immunization. As early as 1936, H. Zinsser immunized guinea pigs with a mixture of live *R*. *prowazekii* and serum from convalescent guinea pigs or immunized horses [173] potentially containing opsonizing or neutralizing antibodies. In this way, he achieved immunity in the treated animals against challenge with *R*. *prowazekii*. One month after immunization, the animals were still immune against the bacteria. Another example is the immunization of humans with a low-virulence strain of *O*. *tsutsugamushi*, which induced solid protection [174]. Similarly, the infection with *O*. *tsutsugamushi* followed by early antibiotic treatment resulted in protection against the homologous strain [175,176].

A safer possibility of immunization may be the use of avirulent or attentuated rickettsiae. A human isolate of *R*. *prowazekii* that was obtained during the second World War and passaged several times in embryonated chicken eggs turned out to be of low virulence. Vaccination with this strain (Madrid E) has been tested in prisoner volunteers in the Mississippi State Prison and was found to protect humans against the infection with a virulent *R*. *prowazekii* strain and to confer long-term immunity. The vast majority of vaccinated people was still protected against the infection with virulent *R*. *prowazekii* up to approximately 5 years [177]. The same avirulent strain *R*. *prowazekii* was also used in field trials in South America and Burundi [178]. The use of avirulent *R*. *prowazekii*, however, bares the risk of reversion to the pathogenic form. Virulence of *R*. *prowazekii* Madrid E is steadily increasing after passages in mice and guinea pigs [179], and reversion to the virulent form of *R*. *prowazekii* (Evir) is likely also the reason for the fact that 14% of the people vaccinated with *R*. *prowazekii* Madrid E showed mild illness around 9 to 14 days postimmunization [178]. The loss of virulence of the *R*. *prowazekii* Madrid E strain is a result of a point mutation in the gene encoding for the S-adenosylmethionine-dependent methyltransferase (RP028/RP027) leading to the absence of this enzyme [180], which results in the hypomethylation of surface proteins. Therefore, OmpB of the attenuated Madrid E strain of *R*. *prowazekii* is hypomethylated compared to the same protein from virulent Evir and naturally occuring *R*. *prowazekii* [181]. The virulent reisolate Evir shows a reversion of this mutation and expresses this enzyme again [182].

A more promising and safer way may be the generation of stably attenuated rickettsial strains that are suitable for vaccination by introducing mutations into virulence genes or by deletion of such genes. Although systems for the targeted introduction or deletion of genes in the rickettsial genome are still limited, some have been described. Phopholipase D, which is involved in phagosomal escape of the bacteria, is considered a virulence factor for *R*. *prowazekii* [183], and site-directed knockout of the gene (*pld*) encoding for this enzyme by transformation and homologous recombination resulted in an attenuated strain of *R*. *prowazekii*. Immunization with these bacteria induced protective immunity in guinea pigs against challenge with virulent *R*. *prowazekii* [33]. Other target proteins might be surface proteins that are involved in bacterial adhesion and invasion such as OmpA (only SFG rickettsiae) and OmpB. It has been shown, however, that a targeted knockout of OmpA does not disturb infectivity of *R*. *rickettsii* in the infection of guinea pigs [34]. Here, a LtrA group II intron retrohoming system has been used to insert intronic RNA at the OmpA target site in the rickettsial genome.

The use of attenuated mutant or knockout strains for vaccination is promising, and methods for genetic engineering are evolving. Yet, rickettsial virulence factors that are essential for infectivity and pathogenicity need to be identified.

### Immunization with antigen-presenting cells (APCs) and recombinant antigens

**Fig 2:**
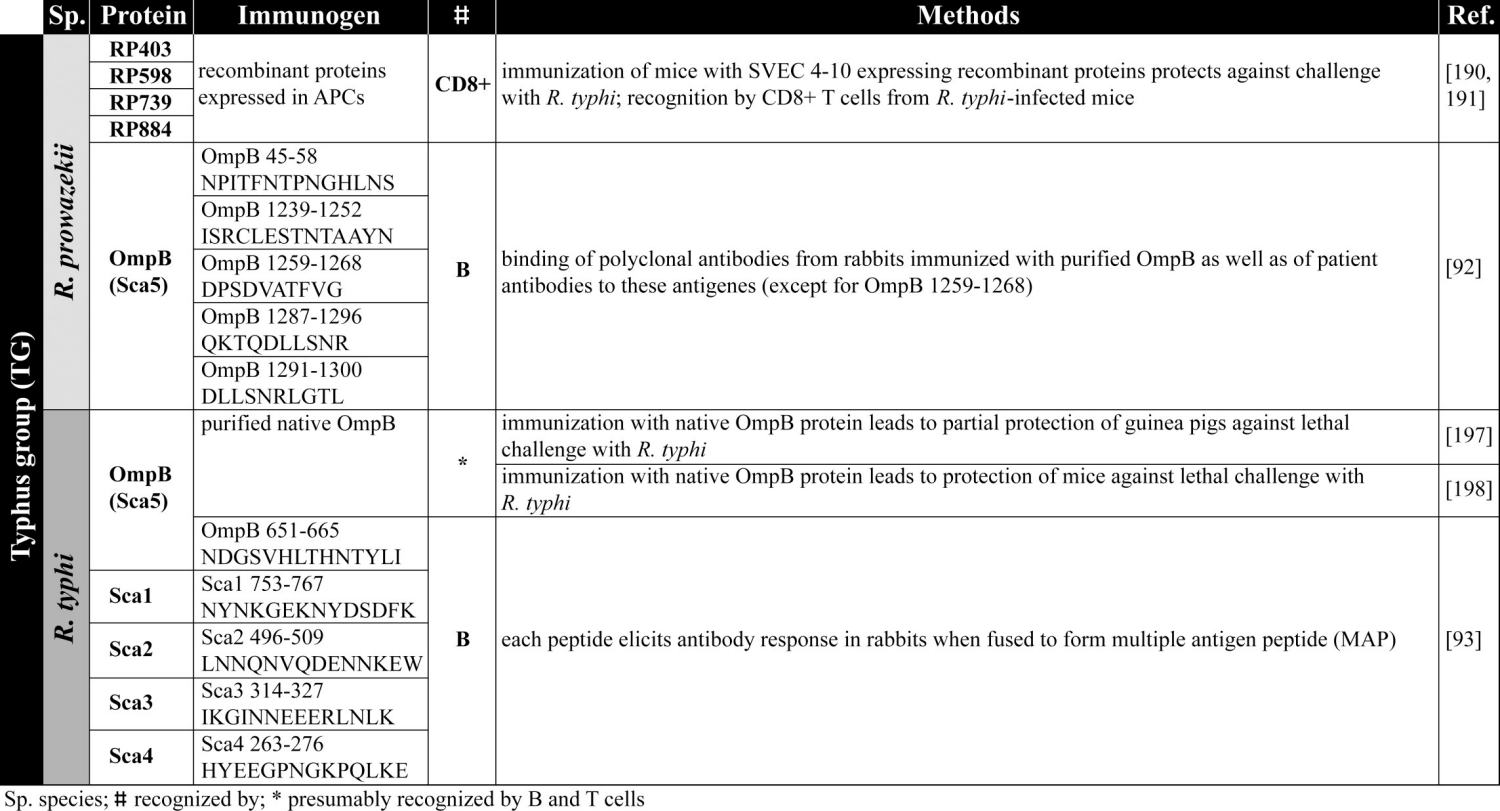
TG antigens. The figure summarizes the antigens identified from TG rickettsiae [92,93,190,191,197,198]. APCs, antigen-presenting cells; MAP, multiple antigen peptide; TG, Typhus group.

**Fig 3:**
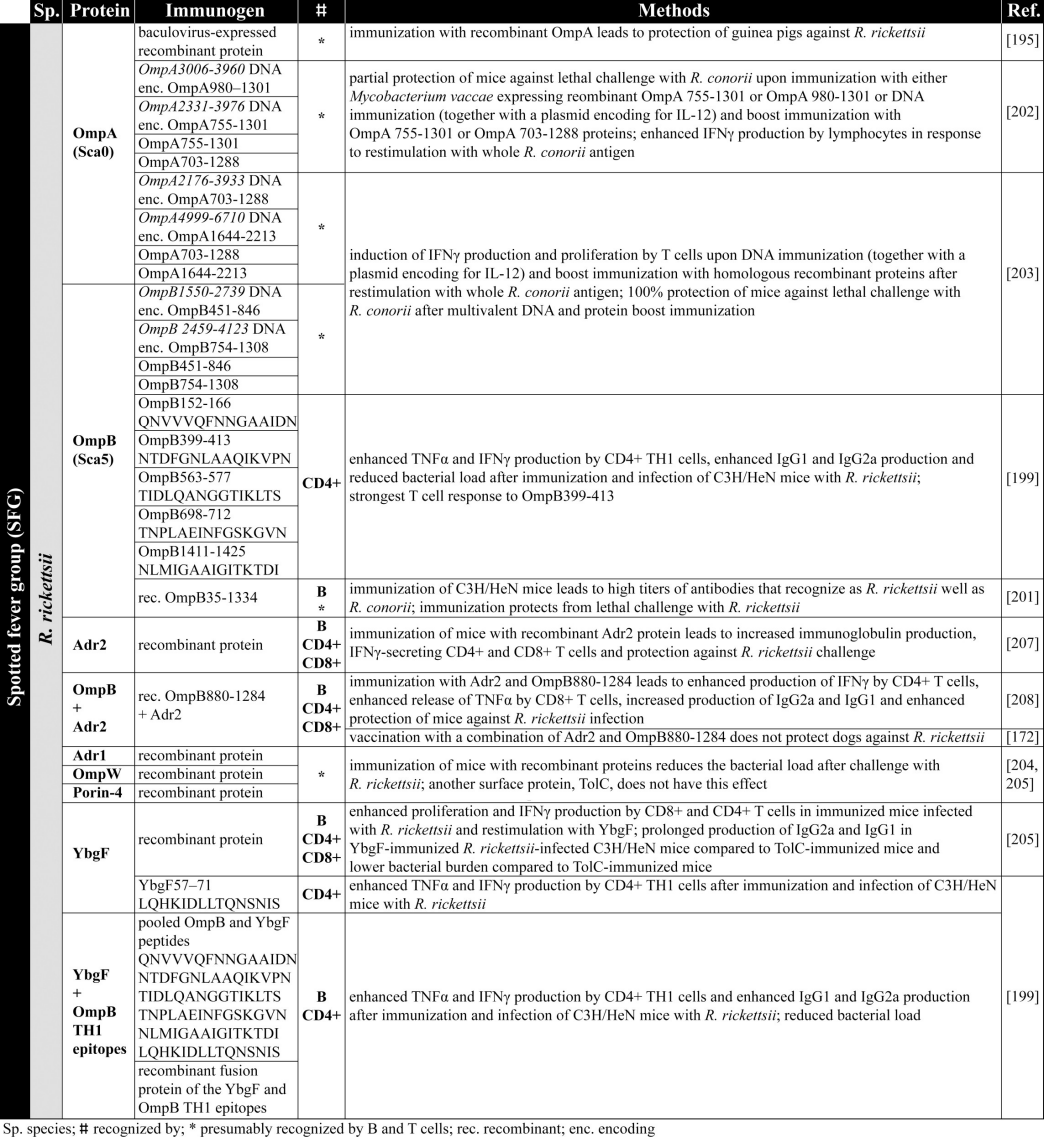
SFG antigens. The figure summarizes the antigens identified from SFG rickettsiae (*R*. *rickettsii*) [172,195,199,201–205,207,208]. enc., encoding; IFNγ, interferon gamma; IgG1, immunoglobulin G1; IgG2a, immunoglobulin G2a; IL-12, interleukin 12; rec., recombinant; SFG, spotted fever group; TNFα, tumor necrosis factor alpha.

**Fig 4:**
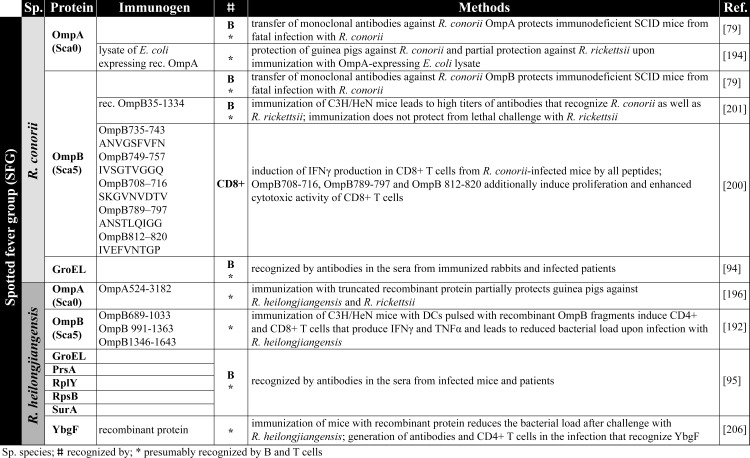
SFG antigens. The figure summerized the antigens identified from SFG rickettsiae (*R*. *conorii* and *R*. *heilongjiangensis*) [79,94,95,192,194,196,200,201,206]. DCs, dendritic cells; IFNγ, interferon gamma; SFG, spotted fever group; TNFα, tumor necrosis factor alpha.

**Fig 5:**
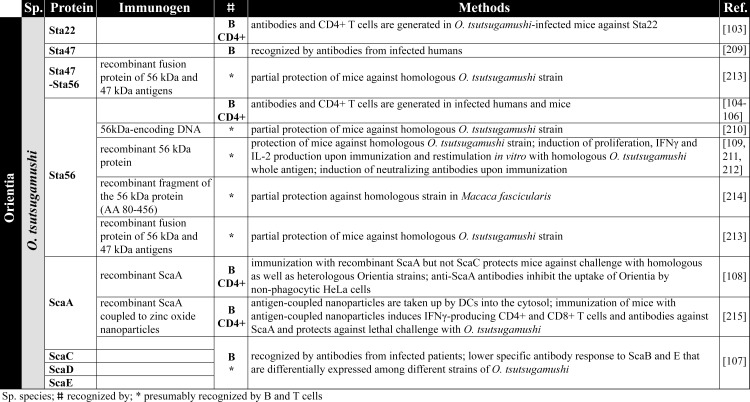
Orientia antigens. The figure summarizes the antigens identified from Orientia [103–109,209–215]. DCs, dendritic cells; IFNγ, interferon gamma; IL-2, interleukin 2.

**Fig 6:**
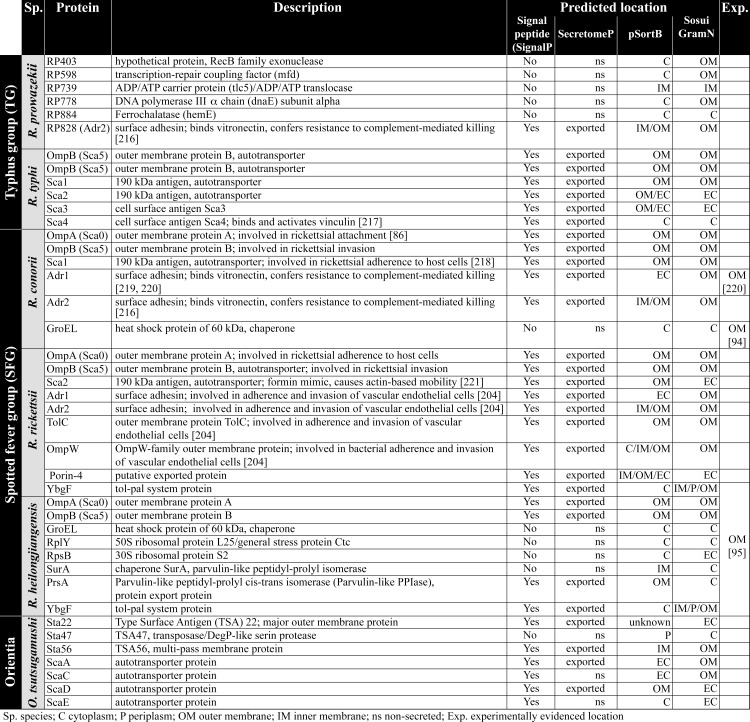
Description of protein function and predicted and/or experimentally evidenced subcellular location. The predicted function and predicted and/or experimentally evidenced location of the immunogenic rickettsial antigens is depicted [86,94,95,204,216–221]. C, cytoplasm; Exp., experimentally evidenced location; IM, inner membrane; ns, non-secreted; OM, outer membrane; P, periplasm; SFG, spotted fever group; Sp., species; TG, typhus group; TSA, Type Surface Antigen.

**Fig 7:**
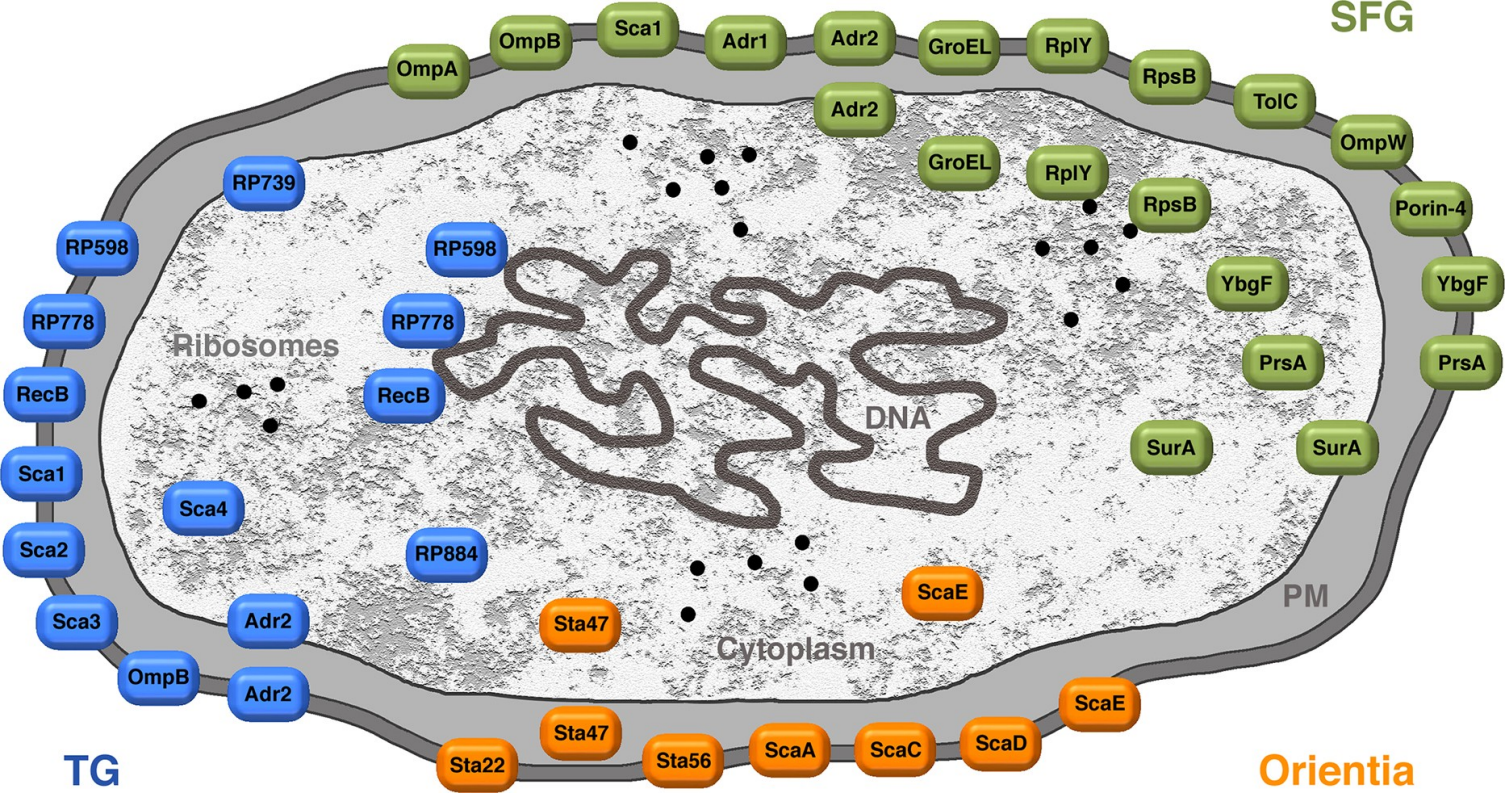
Overview on the antigens identified from Orientia, TG, and SFG rickettsiae and localization of these proteins. The picture provides an overview on the antigens identified from Orientia, TG, and SFG rickettsiae and their subcellular localization. PM, periplasm; SFG, spotted fever group; TG, typhus group.

The safest and most uncomplicated way to achieve immunity against rickettsial infections would be the immunization with recombinant protein antigens or APCs that are capable to induce protective adaptive immune responses. Achievements on this topic of research in recent years as well as different vaccination strategies are summarized below. An overview on rickettsial antigens described in the literature and mentioned in the following chapters is given in Fig 2 (TG antigens), Fig 3 and Fig 4 (SFG antigens), and Fig 5 (Orientia antigens). The supposed function and localization of these proteins within the bacteria is provided in Figs 6 and 7.

#### Immunization with transfected antigen-expressing APCs to activate CD8^+^ T cells

Because CD8^+^ T cells play a dominant role in defense against intracellular rickettsiae, the induction of specific CD8^+^ T cells is a promising way to achieve immunity against rickettsial infections. To induce CD8^+^ T cell responses, antigens have to be directed into the MHC class I presentation pathway, which makes this approach experimentally difficult. Another problem is that rickettsial CD8^+^ T cell antigens and epitopes are still unknown.

Bioinformatic prediction tools (S1 Fig) can be of help for the identification of immunogenic proteins and epitopes. Employing such bioinformatic algorithms, Gazi and colleagues and Caro-Gomez and colleagues identified for the first time CD8^+^ T cell antigens of *R*. *prowazekii*. They analyzed 834 proteins from *R*. *prowazekii* for 9mer peptides that can be presented in MHC class I H-2K^K^ molecules using NetMHCpan, IEBD-Ann, and SYFPEITHI [184–187]. The proteins identified with these methods were further analyzed with RANKPEP, an algorithm that evaluates both MHC class I binding affinity and proteasome processing [188], Vaxign and Vaxitope [189]. Using these bioinformatic approaches, they identified 5 proteins from *R*. *prowazekii* (RP403, RP598, RP739, RP778, and RP884) that may be recognized by CD8^+^ T cells. They further expressed these proteins in SVEC4-10 ECs. These cells derive from C3H/HeJ mice and express the costimulatory molecules CD137L and CD80, facilitating T cell activation. In this way, MHCI presentation was achieved, and the cells were further used as APCs for the immunization of C3H/HeN mice followed by a lethal challenge with *R*. *typhi*. Immunization of the mice prior to the infection with *R*. *typhi* led to increased production of IFNγ and Granzyme B by CD8^+^ T cells and protected the mice from lethal outcome [190,191]. Furthermore, immunization of C3H/HeN mice with SVEC4-10 cells expressing a mixture of these antigens even led to partial protection against a lethal challenge with *R*. *conorii* [191]. Thus, these findings not only demonstrate that these antigens are recognized by CD8^+^ T cells but also that immunization with these proteins can confer cross-protection between the 2 TG rickettsiae as well as SFG rickettsiae, at least in part.

#### Immunization with antigen-pulsed APCs

Another approach for the use of APCs for immunization is to feed the cells with protein antigen. Going this way, one would expect that the proteins are taken up by the APCs to be processed and presented in the context of MHCII leading predominantly to the induction of CD4^+^ T cells.

Meng and colleagues pulsed DCs with different overlapping recombinant OmpB fragments (OmpB_371-702_, OmpB_689-1033_, OmpB_991-1363_, and OmpB_1346-1643_) from *R*. *heilongjiangensis* and subsequently adoptively transferred the DCs into C3H/HeN mice. Mice that were immunized in this way with proteins OmpB_689-1033_, OmpB _991–1363_, or OmpB_1346-1643_ were protected against subsequent infection with *R*. *heilongjiangensis* and showed reduced bacterial load, while protein OmpB_371-702_ did not have this effect [192]. In addition, OmpB_689-1033_, OmpB_991-1363_, or OmpB_1346-1643_ but not OmpB_371-702_ induced IFNγ and TNFα expression in CD4^+^ as well as CD8^+^ T cells upon restimulation of T cells from immunized mice with DCs pulsed with the respective OmpB antigen [192], indicating that immunization with DCs pulsed with these antigens leads to the generation of a CD4^+^ T_H_1 and probably cytotoxic CD8^+^ T cell responses. The authors of this study further show that incubation of DCs with all 4 recombinant OmpB fragments or WCA leads to comparable up-regulation of the costimulatory molecules CD40 and CD86 as well as to the up-regulation of MHC class II [192], indicating a stimulatory capacity of the recombinant OmpB fragments and of WCA. Therefore, the differences in the protective capacity and the induction of cytokine production by T cells cannot be explained by differences in the stimulatory capacity of the proteins on the APCs. Important to mention here is that antibodies were generally not generated in mice immunized with pulsed DCs, whether OmpB protein fragments or WCA were used. Thus, antibodies obviously do not play a role in protection in this experimental setup, while an induction of antigen-specific CD4^+^ as well as of CD8^+^ T cells can be achieved.

#### Vaccination with recombinant proteins and peptides

The experimental vaccination strategies mentioned so far are time consuming and expensive and not suitable for large-scale vaccine production. Therefore, it is still an effort to identify antigenic proteins and peptides that can directly be used for the induction of protective immunity. Some candidates are described below.

#### OmpA/OmpB

OmpA and OmpB are considered immunodominant antigens that are recognized by T and B cells from experimentally infected animals as well as from humans suffering from rickettsioses. For example, different OmpB fragments from *R*. *rickettsii* were expressed in THP-1 MΦ, and T cells from patients that were positively tested for the presence of *R*. *rickettsii*, *R*. *typhi*, or *R*. *felis* by PCR/restriction fragment length polymorphism (RFLP) reacted to these cells with enhanced IL-2 and IFNγ production and induced IL-12p70, IL-6, and TNFα in the antigen-expressing THP-1 MΦ [193]. This response is likely to be ascribed mainly to CD8^+^ T cells because expression of the OmpB fragments in the cells results in MHCI presentation. These data also suggest cross-reaction of T cells to conserved epitopes of OmpB from SFG and TG rickettsiae, which makes this protein a promising vaccine candidate for the vaccination against a broad range of rickettsiae.

Regarding OmpA and OmpB, different vaccination strategies have been applied that are described below.

#### *E*. *coli* expressing recombinant OmpA from *R*. *conorii*

There is 1 study where *E*. *coli*-expressing recombinant OmpA from *R*. *conorii*, instead of purified protein, was used for immunization. Immunization of guinea pigs with lysates of these OmpA-expressing *E*. *coli* protected the animals against *R*. *conorii* and partially against *R*. *rickettsii* infection [194]. The underlying mechanisms of protection, however, were not further analyzed.

#### Immunization with recombinant OmpA and OmpB

In most studies, either purified natural rickettsial proteins, recombinant proteins, protein fragments, or peptides were used for immunization. For example, immunization of guinea pigs with baculovirus-expressed purified recombinant *R*. *rickettsii* OmpA was protective against the infection with *R*. *rickettsii* [195] as was the immunization of guinea pigs with truncated OmpA from *R*. *heilongjiangensis* against homologous bacteria and against *R*. *rickettsii* [196]. Guinea pigs and mice were also immunized with purified native OmpB from *R*. *typhi*, which was protective against the infection with this agent [197,198]. For *R*. *prowazekii*, it has been shown that rabbits immunized with recombinant OmpB develop antibodies against this protein, and these were used to identify specific OmpB B cell epitopes that were also recognized by antibodies from human patients [92].

In a recent study, Wang and colleagues identified 5 CD4^+^ T cell epitopes from the OmpB protein of *R*. *rickettsii* (OmpB_152-166_ (QNVVVQFNNGAAIDN), OmpB_399-413_ (NTDFGNLAAQIKVPN), OmpB_563-577_ (TIDLQANGGTIKLTS), OmpB_698-712_ (TNPLAEINFGSKGVN), and OmpB_1411-1425_ (NLMIGAAIGITKTDI)) and 1 peptide from the YbgF protein of *R*. *rickettsii* (YbgF_57–71_ (LQHKIDLLTQNSNIS)). Immunization of C3H/HeN mice with these peptides either alone, pooled, or expressed as a recombinant fusion protein resulted in enhanced expression of IFNγ and TNFα by CD4^+^ T cells as well as increased immunoglobulin G1 (IgG1) and IgG2a production in the infection with *R*. *rickettsii*. Furthermore, immunization with the pooled peptides led to reduced bacterial burden [199].

Also, CD8^+^ T cell epitopes have been identified in the OmpB protein from SFG rickettsiae. Five synthetic peptides of the OmpB protein from *R*. *conorii* (OmpB_708-716_ (SKGVNVDTV), OmpB_789-797_ (ANSTLQIGG), OmpB_812-820_ (IVEFVNTGP), OmpB_735-743_ (ANVGSFVFN), and OmpB_749-757_ (IVSGTVGGQ) induced IFNγ expression by CD8^+^ T cells from *R*. *conorii*-infected C3H/HeN mice upon restimulation in vitro [200]. CD8^+^ T cells that were reactive to OmpB_708-716_, OmpB_789-797_, and OmpB_812-820_ additionally showed enhanced proliferation and cytotoxic activity against *R*. *conorii*-infected SVEC4-10 cells, which were not observed with OmpB_735-743_ and OmpB_749-757_ [200]. Whether immunization with these peptides leads to protective immunity against the infection with *R*. *conorii* or other SFG rickettsiae where these peptides are conserved remains to be investigated. If these peptides can induce protective immunity, it is unlikely that they can mediate cross-protection against TG rickettsiae because the mentioned antigenic OmpB peptides are not expressed by *R*. *prowazekii* and *R*. *typhi* except for OmpB_749-757_.

Cross-protection between SFG rickettsiae has been demonstrated for the vaccination with recombinant OmpA and OmpB fragments. Immunization with *R*. *rickettsii* OmpA and OmB fragments (Fig 3) can effectively induce cross-protection against *R*. *conorii*. Effectiveness and cross-protection employing these proteins, however, differs depending on the species the proteins derive from. Another study shows that vaccination of C3H/HeN mice with recombinant OmpB from *R*. *conorii*, though inducing high titers of antibodies recognizing the protein, was not protective against *R*. *rickettsii*, whereas the immunization with the corresponding OmpB from *R*. *rickettsii* prevented a lethal outcome of the infection with *R*. *conorii* [201]. Thus, the antigenic potential of nearly identical proteins from different rickettsial species may differ.

#### Heterologous prime/boost vaccination

Protective immunization may not only require repeated vaccination with recombinant antigens but different methods of application. A promising approach is heterologous prime-boost vaccination, in which the same antigen is applied by different methods. Few studies describe such attempts employing either bacteria that express recombinant antigen or antigen-encoding DNA for primary vaccination followed by boost immunization with the respective recombinant protein antigen.

Crocquet-Valdes and colleagues showed that immunization of C3H/HeN mice with *Mycobacterium vaccae* that express the DNA encoding for either *R*. *rickettsii* OmpA_980-1301_ or OmpA_755-1301_ followed by boost immunization with recombinant OmpA_755-1301_ or OmpA_703-1288_ fragments leads to partial protection against a lethal outcome of *R*. *conorii* infection [202]. The same was observed when DNA encoding for OmpA_980-1301_ or OmpA_755-1301_ was used for primary immunization and boost immunization with the before-mentioned recombinant proteins [202]. The authors further observed that lymphocytes from mice immunized with the mentioned DNAs followed by boost immunization with OmpA_980-1301_ or OmpA_755-1301_ protein produced enhanced levels of IFNγ upon restimulation with *R*. *conorii* WCA in vitro, demonstrating the recognition of T lymphocyte epitopes within these fragments. In another work from the same group, mice were repeatedly immunized with DNA encoding for OmpA_703-1288_ or OmpA_1644-6710_ followed by boost vaccination with recombinant OmpA_703-1288_ or OmpA_1644-6710_ protein or with DNA encoding for OmpB_451-846_ or OmpB_754-1308_ followed by boost immunization with recombinant OmpB_451-846_ or OmpB_754-1308_ proteins with a similar outcome [203]. T lymphocytes from immunized mice in this way produced IFNγ upon restimulation with OmpB fragments OmpA_703-1288_ and OmpA_1644-2213_ or OmpB_451-846_ and OmpB_754-1308_, respectively, and 100% of the mice were protected against lethal challenge with *R*. *conorii* upon immunization with mixed plasmid DNA encoding for all 4 protein fragments [203].

Although OmpA and OmpB may be promising candidates for vaccination, it is not clear whether immunization against these proteins can indeed confer protection in species other than mice. Very recently, it has been shown that only the immunization with *R*. *rickettsii* WCA but not with a combination of recombinant Adr2 and a recombinant OmpB fragment protected dogs against RMSF [172].

#### Adr1, Adr2, TolC, OmpW, Porin 4, and YbgF

Little is known about the experimental immunization of animals with other potentially antigenic rickettsial proteins. Knowledge about these antigens is therefore combined in this section. Gong and colleagues showed that immunization of C3H/HeN mice with recombinant Adr1, TolC, OmpW, or Porin 4 from *R*. *rickettsii* results in reduced bacterial load after challenge with homologous bacteria [204], and immunization of C3H/HeN mice with recombinant *R*. *rickettsii* YbgF protein leads to enhanced proliferation and IFNγ production by CD8^+^ and CD4^+^ T cells from *R*. *rickettsii*-infected mice upon in vitro restimulation with YbgF [205]. Similiarly, Qi and colleagues found that YbgF is recognized by CD4^+^ T cells from *R*. *heilongjiangensis-*infected C3H/HeN mice. In addition, antibodies are generated against this protein, and the immunization with YbgF leads to reduced bacterial burden upon challenge with *R*. *heilongjiangensis* [206].

Apart from Adr1, Adr2 also has immunogenic potential. Recombinant Adr2 from *R*. *rickettsii* was used for the immunization of C3H/HeN mice and found to be protective against the infection [207]. CD4^+^ and CD8^+^ from *R*. *rickettsii*-infected animals produced higher levels of IFNγ and showed increased antibody production upon prior immunization recombinant with Adr2 [207], both of which may contribute to protection. Similarly, vaccination of C3H/HeN mice with a combination of *R*. *rickettsii* Adr2 and a fragment of OmpB leads to enhanced IFNγ production by CD4^+^ T cells and TNFα release by CD8^+^ T cells, increased IgG2a and IgG1 generation, and enhanced protection against *R*. *rickettsii* [208].

Adr1, Adr2, TolC, OmpW, Porin 4, and YbgF therefore might represent promising vaccine candidates apart from OmpA and OmpB, although the immunogenicity of these proteins and the immune reactions that are induced have to be further investigated.

#### *O*. *tsutsugamushi* Sta22, Sta47, Sta56, ScaA, ScaC, ScaD, and ScaE

*O*. *tsutsugamushi* phylogenetically differs from other rickettsiae and represents a unique genus of *Rickettsiceae*. For *O*. *tsutsugamushi*, other immunogenic proteins have been described, namely Sta22, Sta47, and Sta56. *O*. *tsutsugamushi*-infected mice develop antibodies and specific CD4^+^ T cells against Sta22 [103]. The same is true for *O*. *tsutsugamushi*-infected humans. Humans additionally develop Sta56-specific antibodies and CD4^+^ T cells [104–106] as well as antibodies recognizing Sta47 [209]. Further investigations focused on the Sta47 and Sta56 proteins. The immunization of mice with DNA encoding for Sta56 was partially protective against the infection with the homologous *O*. *tsutsugamushi* strain [210], and mice vaccinated with recombinant Sta56 protein were found to be completely protected against challenge with the homologous *O*. *tsutsugamushi* strain [109,211,212]. In these animals, enhanced levels of antibodies were observed, and lymphocytes showed increased proliferation, IFNγ, and IL-2 release upon restimulation in vitro with homologous *O*. *tsutsugamushi* WCA [109,211]. Similarly, vaccination of mice with a fusion protein of Sta46 and Sta56 was partially protective against challenge with homologous *O*. *tsutsugamushi* strain [213]. However, only weak protection was achieved in primates (*Macaca fascicularis*) upon immunization with a recombinant fragment of Sta56 (AA 80–456). All of these animals developed fever and rickettsiemia [214].

Other immunodominant antigens are ScaA, C, D, and E from *Orientia*. *O*. *tsutsugamushi* patients develop antibodies against these surface proteins with a stronger response to ScaA and C compared to Sca E and D that are differentially expressed by different *O*. *tsutsugamushi* strains [107]. In the literature, there is 1 description in which C57BL/6 mice were immunized with purified recombinant ScaA, ScaC, or Sta56 that derived from the *O*. *tsutsugamushi* Boryong strain. Immunization with ScaA but not ScaC or Sta56 led to protection and enhanced survival of the animals upon infection with the homologous *O*. *tsutsugamushi* strain. In addition, immunization with recombinant ScaA from the strain Boryong also led to enhanced survival upon infection with the Karp strain as well as in the infection with the Kato strain, although to weaker extent [108]. In another study from the same group, immunization was performed with antigens coupled to nanoparticles. Ha and colleagues coupled ScaA from *O*. *tsutsugamushi* to zinc oxide nanoparticles. These particles are taken up by DCs in vitro and induce protective immunity in vivo in mice. Animals vaccinated with the ScaA-coupled nanoparticles were protected against lethal challenge with *O*. *tsutsugamushi* and developed antibodies against ScaA as well as IFNγ-producing CD4^+^ T_H_1 and CD8^+^ T cells similar to the vaccination of mice with ScaA plus adjuvants [215].

## Concluding remarks

In recent years, few but promising vaccination strategies against rickettsial infections in experimental animal models have been described. OmpA and OmpB are the most prominent antigens that may serve as vaccine candidates, although it is not yet clear whether immunization with these proteins can indeed confer protection. The observations made in immunized and experimentally infected animals, however, are encouraging that the development of a vaccine is possible. Apart from these proteins, only very few other rickettsial antigens have been described. Further research should focus on the idenfication of new rickettsial antigens and the analysis of their immunogenic potential. This research is essential for the development of a protective vaccine that can serve as a prophylaxis against rickettsial infections in endemic areas that are predominantly found in poor countries, as well as for travelers of these regions.

## Supporting information

S1 Fig A selection of bioinformatic software tools for the prediction of cellular protein location, immunogenicity, MHC processing, and B cell epitopes. The table provides a selection of bioinformatic software tools for the prediction of cellular protein location, immunogenicity, processing for MHCI, or MHCII presentation and B cell epitopes.
(PDF)
