# Supplementary material for: The neglected challenge: Vaccination against rickettsiae
Source: PLoS Negl Trop Dis. 2020 Oct 22;14(10):e0008704. doi: 10.1371/journal.pntd.0008704 (PMC7581008; doi:10.1371/journal.pntd.0008704)
Supplement: S1 Fig — The table provides a selection of bioinformatic software tools for the prediction of cellular protein location, immunogenicity, processing for MHCI, or MHCII presentation and B cell epitopes. (PDF) [file pntd.0008704.s001.pdf]

S1 Fig

Name	Server adress	Function	Ref.
**Protein localization**			
SOSUIGramN	http://harrier.nagahama-i-bio.ac.jp/sosui/sosuigramn/sosuigramn_submit.html	subcellular localization of proteins in Gram-negative bacteria	[1]
pSORTb	https://psort.org/	subcellular localization of proteins in Gram-negative bacteria	[2]
SignalP	http://www.cbs.dtu.dk/services/SignalP/	predicts signal peptides and the location of their cleavage sites in proteins from Archaea, Gram-positive Bacteria, Gram-negative Bacteria and Eukarya	[3]
SecretomeP	http://www.cbs.dtu.dk/services/SecretomeP/	predicts non-classical *i.e.* not signal peptide triggered protein secretion	[4]
Phobius	https://www.ebi.ac.uk/Tools/pfa/phobius/	preediction of transmembrane topology and signal peptides	[5]
LipoP	http://www.cbs.dtu.dk/services/LipoP/	predictions of lipoproteins; discriminates between lipoprotein signal peptides, other signal peptides and N-terminal membrane helices in Gram-negative bacteria	[6]
**Immunogenicity, MHC presentation and B cell epitopes**			
**Name**	**Server adress**	**Function**	**Ref.**
VaxiJen	http://www.ddg-pharmfac.net/ vaxijen/VaxiJen/VaxiJen.html	prediction of protective antigens and subunit vaccines	[7]
Vaxign	http://www.violinet.org/vaxign/index.php#dvq http://www.violinet.org/vaxign/index.php#dva	vaccine target prediction and analysis system based on the principle of reverse vaccinology	[8]
PRED$^{BALB/c}$	http://cvc.dfci.harvard.edu/balbc/ http://antigen.i2r.a-star.edu.sg/predBalbc/	prediction of peptides binding to all five MHC molecules in BALB/c mice (class I H2-K^{d}, H2-L^{d}, H2-D^{d} and class II I-A^{d} and I-E^{d})	[9]
PRED(TAP)	http://antigen.i2r.a-star.edu.sg/predTAP	prediction of protein binding to the human transporter associated with antigen (TAP) processing	[10]
MHCPred	http://www.ddg-pharmfac.net/mhcpred/MHCPred/	prediction of MHCI and MHCII epitopes (human and mouse)	[11]
NetMHCpan	http://www.cbs.dtu.dk/services/NetMHCpan/	prediction of MHCI-presented epitopes	[12,13]
IEBD-Ann	http://tools.immuneepitope.org /main/html/tcell_tools.html	prediction of MHCI-and MHCII-binding epitopes	[14]
SYFPEITHI	http://www.syfpeithi.de/	prediction of MHCI-binding epitopes	[15]
ANTIGENpro	http://scratch.proteomics.ics.uci.edu	prediction of B cell antigens	[16]
APBpred	http://crdd.osdd.net/raghava/abcpred/	prediction of linear B cell epitopes	[17]
Epitome	http://www.rostlab.org/services/epitome	prediction of B cell epitopes	[18]
IEDB	https://www.iedb.org	prediction of B and T cell epitopes, immunogenicity and antigen processing	[19]

A selection of bioinformatic software tools for the prediction of cellular protein location, immunogenicity, MHC processing and B cell epitopes. The table provides a selection of bioinformatic software tools for the prediction of cellular protein location, immunogenicity, processing

for MHCI or MHCII presentation and B cell epitopes

References

1. Imai K, Asakawa N, Tsuji T, Akazawa F, Ino A, Sonoyama M, et al. SOSUI-GramN: high performance prediction for sub-cellular localization of proteins in gram-negative bacteria. Bioinformation. 2008;2(9):417-21. doi: 10.6026/97320630002417. PubMed PMID: 18795116; PubMed Central PMCID: PMC2533062.

2. Gardy JL, Spencer C, Wang K, Ester M, Tusnady GE, Simon I, et al. PSORT-B: Improving protein subcellular localization prediction for Gram-negative bacteria. Nucleic Acids Res. 2003;31(13):3613-7. doi: 10.1093/nar/gkg602. PubMed PMID: 12824378; PubMed Central PMCID: PMC169008.

3. Almagro Armenteros JJ, Tsirigos KD, Sonderby CK, Petersen TN, Winther O, Brunak S, et al. SignalP 5.0 improves signal peptide predictions using deep neural networks. Nat Biotechnol. 2019;37(4):420-3. doi: 10.1038/s41587-019-0036-z. PubMed PMID: 30778233.

4. Bendtsen JD, Jensen LJ, Blom N, Von Heijne G, Brunak S. Feature-based prediction of non-classical and leaderless protein secretion. Protein Eng Des Sel.. 2004;17(4):349-56. doi: 10.1093/protein/gzh037. PubMed PMID: 15115854.

5. Madeira F, Park YM, Lee J, Buso N, Gur T, Madhusoodanan N, et al. The EMBL-EBI search and sequence analysis tools APIs in 2019. Nucleic Acids Res. 2019;47(W1):W636-W41. doi: 10.1093/nar/gkz268. PubMed PMID: 30976793; PubMed Central PMCID: PMC6602479.

6. Juncker AS, Willenbrock H, Von Heijne G, Brunak S, Nielsen H, Krogh A. Prediction of lipoprotein signal peptides in Gram-negative bacteria. Protein Sci. 2003;12(8):1652-62. doi: 10.1110/ps.0303703. PubMed PMID: 12876315; PubMed Central PMCID: PMC2323952.

7. Doytchinova IA, Flower DR. VaxiJen: a server for prediction of protective antigens, tumour antigens and subunit vaccines. BMC Bioinformatics. 2007;8:4. doi: 10.1186/1471-2105-8-4. PubMed PMID: 17207271; PubMed Central PMCID: PMC1780059.

8. He Y, Racz R, Sayers S, Lin Y, Todd T, Hur J, et al. Updates on the web-based VIOLIN vaccine database and analysis system. Nucleic Acids Res. 2014;42(Database issue):D1124-32. doi: 10.1093/nar/gkt1133. PubMed PMID: 24259431; PubMed Central PMCID: PMC3964998.

9. Zhang GL, Srinivasan KN, Veeramani A, August JT, Brusic V. PREDBALB/c: a system for the prediction of peptide binding to H2d molecules, a haplotype of the BALB/c mouse. Nucleic Acids Res. 2005;33(Web Server issue):W180-3. doi: 10.1093/nar/gki479. PubMed PMID: 15980450; PubMed Central PMCID: PMC1160239.

10. Zhang GL, Petrovsky N, Kwoh CK, August JT, Brusic V. PRED(TAP): a system for prediction of peptide binding to the human transporter associated with antigen processing. Immunome Res. 2006;2:3. doi: 10.1186/1745-7580-2-3. PubMed PMID: 16719926; PubMed Central PMCID: PMC1524936.

11. Guan P, Doytchinova IA, Zygouri C, Flower DR. MHCPred: bringing a quantitative dimension to the online prediction of MHC binding. Appl Bioinformatics. 2003;2(1):63-6. PubMed PMID: 15130834.

12. Nielsen M, Lundegaard C, Worning P, Lauemoller SL, Lamberth K, Buus S, et al. Reliable prediction of T-cell epitopes using neural networks with novel sequence representations. Protein Sci. 2003;12(5):1007-17. doi: 10.1110/ps.0239403. PubMed PMID: 12717023; PubMed Central PMCID: PMC2323871.

13. Nielsen M, Lundegaard C, Worning P, Hvid CS, Lamberth K, Buus S, et al. Improved prediction of MHC class I and class II epitopes using a novel Gibbs sampling approach. Bioinformatics. 2004;20(9):1388-97. doi: 10.1093/bioinformatics/bth100. PubMed PMID: 14962912.

14. Lin HH, Ray S, Tongchusak S, Reinherz EL, Brusic V. Evaluation of MHC class I peptide binding prediction servers: applications for vaccine research. BMC Immunol. 2008;9:8. doi: 10.1186/1471-2172-9-8. PubMed PMID: 18366636; PubMed Central PMCID: PMC2323361.

15. Rammensee H, Bachmann J, Emmerich NP, Bachor OA, Stevanovic S. SYFPEITHI: database for MHC ligands and peptide motifs. Immunogenetics. 1999;50(3-4):213-9. doi: 10.1007/s002510050595. PubMed PMID: 10602881.

16. Magnan CN, Zeller M, Kayala MA, Vigil A, Randall A, Felgner PL, et al. High-throughput prediction of protein antigenicity using protein microarray data. Bioinformatics. 2010;26(23):2936-43. doi: 10.1093/bioinformatics/btq551. PubMed PMID: 20934990; PubMed Central PMCID: PMC2982151.

17. Saha S, Raghava GP. Prediction of continuous B-cell epitopes in an antigen using recurrent neural network. Proteins. 2006;65(1):40-8. doi: 10.1002/prot.21078. PubMed PMID: 16894596.

18. Schlessinger A, Ofran Y, Yachdav G, Rost B. Epitome: database of structure-inferred antigenic epitopes. Nucleic Acids Res. 2006;34(Database issue):D777-80. doi: 10.1093/nar/gkj053. PubMed PMID: 16381978; PubMed Central PMCID: PMC1347416.

19. Vita R, Mahajan S, Overton JA, Dhanda SK, Martini S, Cantrell JR, et al. The Immune Epitope Database (IEDB): 2018 update. Nucleic Acids Res. 2019;47(D1):D339-D43. doi: 10.1093/nar/gky1006. PubMed PMID: 30357391; PubMed Central PMCID: PMC6324067.

References

1. Imai K, Asakawa N, Tsuji T, Akazawa F, Ino A, Sonoyama M, et al. SOSUI-GramN: high performance prediction for sub-cellular localization of proteins in gram-negative bacteria. Bioinformation. 2008;2(9):417-21. doi: 10.6026/97320630002417. PubMed PMID: 18795116; PubMed Central PMCID: PMC2533062.

2. Gardy JL, Spencer C, Wang K, Ester M, Tusnady GE, Simon I, et al. PSORT-B: Improving protein subcellular localization prediction for Gram-negative bacteria. Nucleic Acids Res. 2003;31(13):3613-7. doi: 10.1093/nar/gkg602. PubMed PMID: 12824378; PubMed Central PMCID: PMC169008.

3. Almagro Armenteros JJ, Tsirigos KD, Sonderby CK, Petersen TN, Winther O, Brunak S, et al. SignalP 5.0 improves signal peptide predictions using deep neural networks. Nat Biotechnol. 2019;37(4):420-3. doi: 10.1038/s41587-019-0036-z. PubMed PMID: 30778233.

4. Bendtsen JD, Jensen LJ, Blom N, Von Heijne G, Brunak S. Feature-based prediction of non-classical and leaderless protein secretion. Protein Eng Des Sel.. 2004;17(4):349-56. doi: 10.1093/protein/gzh037. PubMed PMID: 15115854.

5. Madeira F, Park YM, Lee J, Buso N, Gur T, Madhusoodanan N, et al. The EMBL-EBI search and sequence analysis tools APIs in 2019. Nucleic Acids Res. 2019;47(W1):W636-W41. doi: 10.1093/nar/gkz268. PubMed PMID: 30976793; PubMed Central PMCID: PMC6602479.

6. Juncker AS, Willenbrock H, Von Heijne G, Brunak S, Nielsen H, Krogh A. Prediction of lipoprotein signal peptides in Gram-negative bacteria. Protein Sci. 2003;12(8):1652-62. doi: 10.1110/ps.0303703. PubMed PMID: 12876315; PubMed Central PMCID: PMC2323952.

7. Doytchinova IA, Flower DR. VaxiJen: a server for prediction of protective antigens, tumour antigens and subunit vaccines. BMC Bioinformatics. 2007;8:4. doi: 10.1186/1471-2105-8-4. PubMed PMID: 17207271; PubMed Central PMCID: PMC1780059.

8. He Y, Racz R, Sayers S, Lin Y, Todd T, Hur J, et al. Updates on the web-based VIOLIN vaccine database and analysis system. Nucleic Acids Res. 2014;42(Database issue):D1124-32. doi: 10.1093/nar/gkt1133. PubMed PMID: 24259431; PubMed Central PMCID: PMC3964998.

9. Zhang GL, Srinivasan KN, Veeramani A, August JT, Brusic V. PREDBALB/c: a system for the prediction of peptide binding to H2d molecules, a haplotype of the BALB/c mouse. Nucleic Acids Res. 2005;33(Web Server issue):W180-3. doi: 10.1093/nar/gki479. PubMed PMID: 15980450; PubMed Central PMCID: PMC1160239.

10. Zhang GL, Petrovsky N, Kwoh CK, August JT, Brusic V. PRED(TAP): a system for prediction of peptide binding to the human transporter associated with antigen processing. Immunome Res. 2006;2:3. doi: 10.1186/1745-7580-2-3. PubMed PMID: 16719926; PubMed Central PMCID: PMC1524936.

11. Guan P, Doytchinova IA, Zygouri C, Flower DR. MHCPred: bringing a quantitative dimension to the online prediction of MHC binding. Appl Bioinformatics. 2003;2(1):63-6. PubMed PMID: 15130834.

12. Nielsen M, Lundegaard C, Worning P, Lauemoller SL, Lamberth K, Buus S, et al. Reliable prediction of T-cell epitopes using neural networks with novel sequence representations. Protein Sci. 2003;12(5):1007-17. doi: 10.1110/ps.0239403. PubMed PMID: 12717023; PubMed Central PMCID: PMC2323871.

13. Nielsen M, Lundegaard C, Worning P, Hvid CS, Lamberth K, Buus S, et al. Improved prediction of MHC class I and class II epitopes using a novel Gibbs sampling approach. Bioinformatics. 2004;20(9):1388-97. doi: 10.1093/bioinformatics/bth100. PubMed PMID: 14962912.

14. Lin HH, Ray S, Tongchusak S, Reinherz EL, Brusic V. Evaluation of MHC class I peptide binding prediction servers: applications for vaccine research. BMC Immunol. 2008;9:8. doi: 10.1186/1471-2172-9-8. PubMed PMID: 18366636; PubMed Central PMCID: PMC2323361.

15. Rammensee H, Bachmann J, Emmerich NP, Bachor OA, Stevanovic S. SYFPEITHI: database for MHC ligands and peptide motifs. Immunogenetics. 1999;50(3-4):213-9. doi: 10.1007/s002510050595. PubMed PMID: 10602881.

16. Magnan CN, Zeller M, Kayala MA, Vigil A, Randall A, Felgner PL, et al. High-throughput prediction of protein antigenicity using protein microarray data. Bioinformatics. 2010;26(23):2936-43. doi: 10.1093/bioinformatics/btq551. PubMed PMID: 20934990; PubMed Central PMCID: PMC2982151.

17. Saha S, Raghava GP. Prediction of continuous B-cell epitopes in an antigen using recurrent neural network. Proteins. 2006;65(1):40-8. doi: 10.1002/prot.21078. PubMed PMID: 16894596.

18. Schlessinger A, Ofran Y, Yachdav G, Rost B. Epitome: database of structure-inferred antigenic epitopes. Nucleic Acids Res. 2006;34(Database issue):D777-80. doi: 10.1093/nar/gkj053. PubMed PMID: 16381978; PubMed Central PMCID: PMC1347416.

19. Vita R, Mahajan S, Overton JA, Dhanda SK, Martini S, Cantrell JR, et al. The Immune Epitope Database (IEDB): 2018 update. Nucleic Acids Res. 2019;47(D1):D339-D43. doi: 10.1093/nar/gky1006. PubMed PMID: 30357391; PubMed Central PMCID: PMC6324067.
